# Grand-potential based phase-field model for systems with interstitial sites

**DOI:** 10.1038/s41598-020-79956-x

**Published:** 2020-12-30

**Authors:** P. G. Kubendran Amos, Britta Nestler

**Affiliations:** 1 Institute of Applied Materials (IAM-CMS), Karlsruhe Institute of Technology (KIT), Strasse am Forum 7, 76131 Karlsruhe, Germany; 2 Department of Metallurgical and Materials Engineering, National Institute of Technology, Tiruchirappalli, Tamil Nadu 620015 India; 3 Institute of Digital Materials Science (IDM), Karlsruhe University of Applied Sciences, Moltkestr. 30, 76133 Karlsruhe, Germany

**Keywords:** Computational methods, Mechanical engineering

## Abstract

Existing grand-potential based multicomponent phase-field model is extended to handle systems with interstitial sublattice. This is achieved by treating the concentration of alloying elements in site-fraction. Correspondingly, the chemical species are distinguished based on their lattice positions, and their mode of diffusion, interstitial or substitutional, is appropriately realised. An approach to incorporate quantitative driving-force, through parabolic approximation of CALPHAD data, is introduced. By modelling austenite decomposition in ternary Fe–C–Mn, albeit in a representative microstructure, the ability of the current formalism to handle phases with interstitial components, and to distinguish interstitial diffusion from substitutional in grand-potential framework is elucidated. Furthermore, phase transformation under paraequilibrium is modelled to demonstrate the limitation of adopting mole-fraction based formulation to treat multicomponent systems.

## Introduction

Macroscopic properties exhibited by a material are generally dictated by its microstructure. Characteristic features of a microstructure that influence properties include volume fraction of the constituent phases (phase-fraction), and their morphological distribution. Under certain conditions, these microstructural features evolve definitively, thereby establishing a change in the behaviour of the material^1^. In order to comprehensively understand the evolution, the phase transformations and other morphological changes which alters the microstructure are meticulously investigated^2,3^. Considering the intricate three-dimensional distribution of the phases, theoretical techniques, in addition to the regular experimental studies, are adopted to delineate an observed transformation^4^.

A rather conventional theoretical approach to understand microstructural changes involves treating the transformation as a Stefan-like free-boundary problem^5^. Correspondingly, the temporal evolution of an appropriate thermodynamic parameter, like concentration or temperature, is solved under definite free-boundary and Dirichlet (or equilibrium) conditions. The temporal change in the parameter is perceived as the microstructural evolution by monitoring the interface, which is realised by the Dirichlet condition. Given the unambiguous nature of the approach, this ‘sharp-interface’ treatment has considerably been adopted to model phase transformations. Even though transformation kinetics, to a certain degree of accuracy, can be ascertained in the sharp-interface framework, given that the underlying formulation gets increasingly convoluted upon considering the morphology of the phases, this approach is rarely extended to model two- or three-dimensional microstructural changes^6^. In other words, since the microstructural transformations are realised by tracking the migrating-interface, sharp-interface technique demands a sufficiently sophisticated formulation to capture the complex morphology of the evolving phases. Owing to the complexity introduced by the morphological considerations, alternate approaches are adopted to analyse microstructural transformations. Phase-field approach is one such numerical technique which is employed as a potent substitute for sharp-interface treatments^7,8^.

### Phase-field framework

The aspect of the sharp-interface approach, which makes it arduous for modelling phase transformation with intricate morphology, *i*.*e* interface tracking, is circumvented in phase-field technique by introducing a scalar variable, ${\varvec{\phi }}\in \{\phi _{\alpha },\phi _{\beta }\}$. This variable, called phase-field, exhibits a spatio-temporal dependency, ${\varvec{\phi }}(\varvec{x},t)$, and is often held to indicate volume fraction of a phase at given position ($\varvec{x}$), in an instance (*t*)^9^. Attributing volume fraction to phase-field imposes a constraint $\phi _{\alpha }+\phi _{\beta }=1$ at any point in the domain. Consequently, a two phase system, like the ones considered in the present study, can be treated by a single independent scalar variable, $\phi _{\alpha }\equiv \phi$ and $\phi _{\beta }=1-\phi$. There are analogous techniques wherein the constraint associated with the volume fraction are relaxed, which are generally categorised as ‘continuum-field treatments’^10^.

Given that the independent scalar-variable represents the volume fraction of a phase, $\phi =1$ in a spatial position indicates the presence of a phase, say phase-$\alpha$, while $\phi =0$ denotes an absence of it, and thus, characterises phase-$\beta$. Correspondingly, a system of two phases is distinguished as $\{\phi _{\alpha }(\varvec{x},t)\equiv \phi (\varvec{x},t)|\phi (\varvec{x},t)=1\}$ and $\{\phi _{\beta }(\varvec{x},t)\equiv \phi (\varvec{x},t)|\phi (\varvec{x},t)=0\}$. The interface separating the two phases pertains to a diffuse region in the domain, wherein the variable adopts the value $\phi (\varvec{x},t)\in (1,0)$. In other words, the sharp interface separating the phases in the conventional techniques is replaced by a diffuse region in the phase-field treatment, which is characterised by a smooth transition of the scalar variable $\phi (\varvec{x},t)$^11^. This characteristic description of the interface, in the phase-field approach, lends itself to modelling microstructural transformation through the spatio-temporal evolution of the independent scalar-variable, thereby circumventing the arduous task of interface-tracking.

In the phase-field framework, based on its distinctive scalar variable ($\phi$), a system is distinguished into interface and ‘bulk ’ phase, which are respectively characterised by $\phi \in (0,1)$ and $\phi =0,1$. Accordingly, the overall energy-density of the system is described in the form of a functional as

1 $$\begin{aligned} {\mathcal {F}}(\phi , \varvec{\nabla }\phi , \varvec{S})&= {\mathcal {F}}_{\mathrm{int}}(\phi ,\varvec{\nabla }\phi )+{\mathcal {F}}_{\mathrm{bulk}}(\phi ,\varvec{S}) \\&= \int _{V}\underbrace{\left[ f_{\mathrm{int}}(\phi ,\varvec{\nabla }\phi ) + f_{\mathrm{bulk}}(\phi ,\varvec{S}) \right] }_{f(\phi , \varvec{\nabla }\phi , \varvec{S})}\mathrm{d}{V}, \end{aligned}$$

where $f_{\mathrm{int}}(\phi ,\varvec{\nabla }\phi )$ and $f_{\mathrm{bulk}}(\phi ,\varvec{S})$ correspond to the energy contributions of the diffuse interface and bulk phases^12^. Moreover, in Eq. (1), *V* represents the volume of the system. By a reasonable choice of the fundamental parameter with definite, and often well-established, thermodynamic significance, the system under investigation is appropriately described. This parameter which is associated with the bulk contribution ($f_{\mathrm{bulk}}(\phi ,\varvec{S})$), and renders relevant energy-density to the phases, is generically represented by $\varvec{S}$ in Eq. (1). Depending on the nature of the energy-density, the variable $\varvec{S}$ adopts a different form. For instance, when the bulk contribution is elastic-energy density, $\varvec{S}$ depicts stress and/or strain^13,14^, as opposed to scalar temperature (or internal energy-density) in the numerical treatment of solidification^15,16^.

In phase-field modelling, the microstructural transformation reflects the spatio-temporal changes in its characteristic scalar variable, phase-field $\phi (\varvec{x},t)$. Considering that the system migrates towards minimisation of the overall energy-density (${\mathcal {F}}(\phi , \varvec{\nabla }\phi , \varvec{S})$), the evolution of the scalar variable is correspondingly formulated as

2 $$\begin{aligned} \frac{\partial \phi (\varvec{x},t)}{\partial t}&= -{\tilde{M}}\frac{\delta {\mathcal {F}}(\phi , \varvec{\nabla }\phi , \varvec{S})}{\delta \phi } \\&= {\tilde{M}}\left[ \varvec{\nabla }\cdot \frac{\partial f(\phi , \varvec{\nabla }\phi , \varvec{S})}{\partial \varvec{\nabla }\phi } - \frac{\partial f(\phi , \varvec{\nabla }\phi , \varvec{S})}{\partial \phi }\right] , \end{aligned}$$

where ${\tilde{M}}$ dictates the mobility of phase-field. The contribution of the bulk phases to the spatio-temporal change in Eq. (2) is augmented by solving for the evolution of the fundamental variable, $\varvec{S}$. Depending on the thermodynamic nature of this variable, the expression governing its evolution varies.

### Multicomponent models

#### Single-phase approach

Phase-field technique handling microstructural changes in alloys with more than one independent components can be succinctly reviewed by considering the nature of fundamental variable employed to formulate the bulk contribution. Pioneering attempts to model phase transformation in multicomponent system adopt ‘homogeneous’ concentration, expressed as

3 $$\begin{aligned} \varvec{S}\equiv \varvec{c}(\varvec{x},t)=\{c_1,c_2,\dots ,c_{m}\}, \end{aligned}$$

to describe energy-density of the individual phases^17,18^. In this approach, as indicated by Eq. (3), the fundamental variable adopts a form of a tuple with *m* entities, wherein *m* is the total number of chemical species in the system, and $c_m$ represents the concentration of component-*m* in mole-fraction. This concentration, $\varvec{c}(\varvec{x},t)$, through its spatio-temporal evolution influences the bulk contribution, which ultimately governs the phase transformation. Since the spatially-varying concentration at the diffuse interface can be viewed as the composition of a single phase comprising (or coarse-grained) of the adjoining bulk phases, the treatment is referred to ‘single-phase’ or ‘coarse-graining’ approach^19,20^.

Subsequent works adopting the single-phase approach realised that the choice of the fundamental variable shown in Eq. (3), inherently couples the bulk and interface contribution^21^. In other words, the free-energy densities of phases formulated through the homogeneous concentration $\varvec{c}(\varvec{x},t)$ influences the features of the diffuse interface including its energy-density and width^22^. Owing to this limitation of the single-phase models, alternate techniques have been formulated wherein the bulk and interface contributions are effectively decoupled.

#### Two-phase approach

The restriction imposed by the bulk contribution on the diffuse interface is generally averted in the subsequent phase-field approaches by employing phase-dependent ‘fictitious’ concentrations^23^. This fundamental dynamic variable, which lends itself to the decoupling of the bulk and interface contribution, is written as

4 $$\begin{aligned} \varvec{S}\equiv \{\varvec{c^{\alpha }},\varvec{c^{\beta }}\}=\{(c^{\alpha }_1,c^{\alpha }_2,\dots ,c^{\alpha }_{m}),(c^{\beta }_1,c^{\beta }_2,\dots ,c^{\beta }_{m})\}, \end{aligned}$$

where $\varvec{c^{\alpha }}$ and $\varvec{c^{\beta }}$ are *m*-tuple phase-dependent concentration pertaining to phase-$\alpha$ and -$\beta$, with each entries denoting mole-fraction of an individual component in the corresponding phase^24,25^. In contrast to the single-phase approach, since the interface in this technique comprises of two distinct fictitious concentrations, with each associated to an individual phase, it is referred to as ‘two-phase ’ model^19,20^.

Although the introduction of the fictitious concentrations, $\varvec{c^{\alpha }}$ and $\varvec{c^{\beta }}$, avoids any coupling of the bulk and interface contribution, the overall computational burden is increased in this formulation. In other words, since the phase-dependent concentrations, which fundamentally describe the energy-density of the phases, are ‘previously unknown’ , additional resources are spent to ascertain them at each instance (time-step) of the evolution. With the increase in the number of independent components, this computational effort gets additionally confounded.

In systems with dilute compositions, the fictitious concentrations are realised through ‘partitioning coefficient’, which is the ratio of the equilibrium concentration of the constituent phase-$\alpha$ and -$\beta$^21^. On the other hand, generally, the phase-dependent concentrations are determined by assuming a quasi-equilibrium condition at the interface^24^. The quasi-equilibrium condition in the two-phase formulation that lends itself to the estimation of phase-dependent concentrations is expressed as

5 $$\begin{aligned} \mu _{i}^{\alpha }(\varvec{x},t)=\mu _{i}^{\beta }(\varvec{x},t)\equiv \mu _{i}(\varvec{x},t) \qquad \forall \; i\in \{2,\dots ,m\}, \end{aligned}$$

where $\mu _{i}(\varvec{x},t)$ denotes the *diffusion potential* of component-*i*, which is the difference in the chemical potential of species-*i* and matrix ($i=1$)^25^. Based on the local-equivalence of the diffusion potential (Eq. (5)), the phase-dependent concentrations are calculated through a continuity (interpolated) relation involving phase-field.

#### Grand-potential approach

In order to curtail the computational burden imposed by the phase-dependent concentrations, an alternate model is devised, wherein a single continuous variable is adopted to describe the bulk contribution. Despite its similarity to the single-phase approach, this model elegantly decouples bulk and interface contribution by treating diffusion potential as the dynamic variable^19^. The change in the fundamental variables from concentration to diffusion potential is achieved by the Legendre transformation of the free-energy density. Considering that the Legendre transformation of the free-energy density yields the grand-chemical potential of phases, this technique is referred to ‘grand-potential’ model^19,26,27^. The fundamental variables of grand-potential technique are expressed as

6 $$\begin{aligned} \varvec{S}\equiv \{\varvec{c^{\alpha }}(\varvec{\mu }),\varvec{c^{\beta }}(\varvec{\mu })\}, \end{aligned}$$

where the diffusion potential represented as a tuple of $m-1$ entities that read

7 $$\begin{aligned} \varvec{\mu }=\{\mu _2,\dots ,\mu _{m}\}. \end{aligned}$$

In grand-potential formalism, the phase-dependent concentrations are ascertained based on the *invertibility* of diffusion-potential and concentration. This technique of determining the fictitious concentrations is claimed to comparatively reduce the overall computational cost.

### Limitations of conventional formalism

Despite the differences in the choice of fundamental dynamic variables, one similarity is rather evident in all multicomponent phase-field models discussed briefly in previous “Multicomponent models” section. In all these approaches, mole-fraction is largely employed to represent, and handle, the concentration of the various chemical components. Mole-fraction, in addition to being inherently dimensionless, elegantly facilitates the realisation of dependent and independent concentration variables. The characteristic summation relation, $c_{1}^{\alpha }+\sum _{i=2}^{m}c_{2}^{\alpha }=1$, which stems from the description of mole-fraction, is readily exploited to identify $m-1$ independent variables in a system of *m*-components. Even though, there are certain advantages in preferring mole-fraction, particularly, in theoretical treatments, the restriction imposed by this choice of representing concentrations is noticeable, and cannot entirely be overlooked^28^.

The characteristic summation relation of the mole-fraction is adopted to estimate the evolution of the dependent variable based on the corresponding spatio-temporal change in the independent concentrations. Such treatment implicitly assumes that the diffusion is ‘substitutional’ in nature. In other words, the evolution of the independent components warrants a reverse flow of the dependent variable to ensure the recovery of the summation constraint. Although the complementing reverse-flow of the dependent concentration, often matrix-species, is consistent with substitutional diffusion, similar evolution accompanying the diffusion of interstitial components is counterintuitive, and fundamentally contradicts the perception of interstitial migration. For instance, the mole-fraction based numerical treatment of alloying-element diffusion in Fe–C and Fe–Mn system would render an identical evolution of matrix-Fe, thereby explicating the inconsistency associated with this approach. Furthermore, owing to the implicit coupling of the concentration of matrix to all other alloying-elements, the evolution under definite equilibrium condition like para- and constrained carbon-equilibrium, wherein certain species migrate without largely altering the local composition of other components, cannot be modelled in a framework based on mole-fraction^29^.

### Existing alternate approaches

The restriction primarily imposed by the mole-fraction based treatment of concentration in multicomponent phase-field models have already been identified, and elaborately discussed^28^. Alternate techniques that circumvent this constraint, and facilitate the incorporation of interstitial diffusion, treat concentration in molar number-density (mol/m$^3$), wherein number of moles per unit volume is considered^28–30^. In these formulations, two assumptions are critically made concerning the partial molar-volume of the components:

- All components occupying the regular-lattice positions are considered to render equal contribution to the volume
- Influence of interstitial chemical-species on volume of the phase is negligible

In other words, depending on the lattice positions, the components are considered to have either identical or zero partial molar-volume. This consideration inherently distinguishes the components that evolves substitutionally from the interstitially migrating ones, thereby relaxing the constraint of mole-fraction based approaches.

In addition to molar number-density based models, an exhaustive approach involving site-fraction has been reported^31^. The ability of this technique extends beyond realising substitutional and interstitial components to distinguishing different complex sublattices in a system. Coupling site-fraction based CALPHAD data to incorporate quantitative driving-force is comprehensively discussed in this work^31^.

Existing phase-field models, wherein molar number-density or site-fraction is adopted to represent and handle concentrations, are formulated either in single-^28,29^ or two-phase framework^30,31^ discussed in “Single-phase approach” and “Two-phase approach” sections. Consequently, the computational burden associated with these approaches is inherently carried-over to these rather sophisticated number-density or site-fraction based techniques. A grand-potential model employing molar number-density has recently been reported by the authors of the current work, as a numerically efficient alternate for the existing formalisms^20^. Since this re-visited approach adopts the conventional assumptions of negligible and identical partial molar-volume of interstitial and regular-lattice components, a multi-component grand-potential model involving site-fraction is presented here.

The fundamental variables of the site-fraction based grand-potential technique is written as

8 $$\begin{aligned} \varvec{S}\equiv \{\varvec{y^{\alpha }}(\varvec{\mu }),\varvec{y^{\beta }}(\varvec{\mu })\}, \end{aligned}$$

where $\varvec{y^{\alpha }}$ and $\varvec{y^{\beta }}$ are tuples with $m-1$-entries with each representing the concentration of individual components in site-fraction. The tuple $\varvec{\mu }$ encompasses the diffusion- and chemical-potential of regular and interstitial components. This aspect of the dynamic variable $\varvec{\mu }$ will be discussed in the subsequent sections.

## Multicomponent grand potential model

### Site- and mole-fraction

Concentration of a component-*i* in phase-$\Theta '$ with *s* sublattices is related to the appropriate site-fractions through the relation

9 $$\begin{aligned} c_{i}^{\Theta '}=\frac{\sum _{\mathrm{s}}N_{i}^{\Theta '}}{\sum _{\mathrm{s}}\sum _{j}N_{\mathrm{s}:j}^{\Theta '}} = \frac{\sum _{\mathrm{s}}{\tilde{a}}_{\mathrm{s}}^{\Theta '}y_{\mathrm{s}:i}^{\Theta '}}{\sum _{\mathrm{s}}{\tilde{a}}_{\mathrm{s}}^{\Theta '}(1-y_{\mathrm{s}:va}^{\Theta '})}, \end{aligned}$$

where $N_{i}^{\Theta '}$ is the number of moles of component-*i* in all of phase-$\Theta '$, while number of moles of *j* in sublattice-*s* is denoted by $N_{s:j}^{\Theta '}$. Site-fraction of component-*i* and vacancy in the sublattice-s is correspondingly denoted by $y_{\mathrm{s}:i}^{\Theta '}$ and $y_{\mathrm{s}:va}^{\Theta '}$. In Eq. (9), ${\tilde{a}}_{\mathrm{s}}^{\Theta '}$ introduces all sites available in sublattice-*s* of phase-$\Theta '$.

Existing phase-field approach, wherein systems with multiple sublattices are handled, Eq. (9) is employed to relate site- and mole-fraction^31^. However, the present consideration of systems with regular and interstitial lattice, lends itself to a rather straightforward treatment of concentrations.

Firstly, the overall composition of a phases ($\Theta \in \{\alpha ,\beta \}$) can simply be expressed as

10 $$\begin{aligned} \varvec{y}^{\Theta }=\{\underbrace{y_{\mathrm{sub}:1}^{\Theta }, y_{\mathrm{sub}:2}^{\Theta }, \dots , y_{\mathrm{sub}:j}^{\Theta }}_{\varvec{y}^{\Theta }_{\mathrm{sub}}}, \underbrace{y_{\mathrm{ints}:1}^{\Theta }, y_{\mathrm{ints}:2}^{\Theta }, \dots , y_{\mathrm{ints}:l}^{\Theta }}_{\varvec{y}^{\Theta }_{\mathrm{ints}}},y_{\mathrm{ints}:va}^{\Theta }\}, \end{aligned}$$

where $\varvec{y}^{\Theta }_{\mathrm{sub}}$ and $\varvec{y}^{\Theta }_{\mathrm{ints}}$ represent the *j*- and *l*(+1)-tuple comprising of site-fractions of components occupying regular and interstitial sublattices, respectively. Moreover, $y_{\mathrm{sub}:j}^{\Theta }$ and $y_{\mathrm{ints}:l}^{\Theta }$ correspond to the site-fractions of regular and interstitial component-*j* and -*l* in their corresponding lattice, with $y_{\mathrm{ints}:va}^{\Theta }$ denoting the concentration of vacancies in interstitial sublattice.

Secondly, a more elegant relation, when compared to Eq. (9), can be derived between site- and mole-fraction by introducing a constant, $a^{\Theta }$. This phase-dependent constant, $a^{\Theta }$, is the ratio of the total number of interstitial and regular-lattice sites, and is written as

11 $$\begin{aligned} a^{\Theta } = \frac{\sum _{k=1}^{l}N_{\mathrm{ints}:k}^{\Theta } + N_{\mathrm{ints}:va}^{\Theta }}{\sum _{i=1}^{j}N_{\mathrm{sub}:i}^{\Theta }}, \end{aligned}$$

where $N_{\mathrm{ints}:k}^{\Theta }$ represents the number of moles of interstitial sites that are occupied by alloying element-*k*, and the vacant sites in corresponding sublattice are denoted by $N_{\mathrm{ints}:va}^{\Theta }$. Furthermore, assuming that regular lattice-sites are completely occupied, it is quantified as $\sum _{i=1}^{j}N_{\mathrm{sub}:i}^{\Theta }$ in Eq. (11).

Owing to its atomic size, a chemical species is generally restricted to one sublattice, and almost never occupies a non-similar site^32^. In other words, a component-*i* associated with the regular lattice, due to the largeness of its atomic size, does not occupy interstitial site. Additionally, it is important to realise that in Eq. (11), as in Eq. (10), the interstitial lattice is distinguished into vacant and occupied sites, consequently, the total number of interstitial sites in phase-$\Theta$ is represented by $\sum _{k=1}^{l}N_{\mathrm{ints}:k}^{\Theta } + N_{\mathrm{ints}:va}^{\Theta }$.

As opposed to mole-fraction, that wholly focuses on expressing the concentration of an individual species, irrespective of its spatial position, site-fraction describes the relative amount of an alloying element present in a given sublattice. Accordingly, site-fraction of component-*k* which is exclusively associated with the interstitial lattice of phase-$\Theta$ is defined, and subsequently, related to its corresponding mole-fraction ($c_{\mathrm{ints}:k}^{\Theta }$) as

12 $$\begin{aligned} y_{\mathrm{ints}:k}^{\Theta }&= \frac{N_{\mathrm{ints}:k}^{\Theta }}{\sum _{k=1}^{l}N_{\mathrm{ints}:k}^{\Theta }+N_{\mathrm{ints}:va}^{\Theta }} \\&= c_{\mathrm{ints}:k}^{\Theta }\left[ \frac{\sum _{i=1}^{j}N_{\mathrm{sub}:i}^{\Theta }+\sum _{k=1}^{l}N_{\mathrm{ints}:k}^{\Theta }}{\sum _{k=1}^{l}N_{\mathrm{ints}:k}^{\Theta }+N_{\mathrm{ints}:va}^{\Theta }} \right] . \end{aligned}$$

Using constant $a^{\Theta }$, introduced in Eq. (11), the above expression is simplified as

13 $$\begin{aligned} y_{\mathrm{ints}:k}^{\Theta }=\frac{1}{a^{\Theta }}\left[ \frac{c_{\mathrm{ints}:k}^{\Theta }}{1-\sum _{k=1}^{l} c_{\mathrm{ints}:k}^{\Theta }}\right] \left( = \frac{1}{a^{\Theta }}\left[ \frac{c_{\mathrm{ints}:k}^{\Theta }}{\sum _{i=1}^{j} c_{\mathrm{sub}:i}^{\Theta }}\right] \right) . \end{aligned}$$

This relation, though applicable only to systems with regular and interstitial sublattices, can directly be adopted to express concentration in site-fraction, provided the site-ratio constant $a^{\Theta }$ is accurately estimated.

In its current form, $a^{\Theta }$ is hugely dictated by the crystal structure of phase-$\Theta$, and based on the periodic arrangement of the chemical species, this site-ratio constant can be suitably ascertained. Moreover, as will be evident in the subsequent sections, it is possible to incorporate stoichiometry between alloying elements through the ratio constant $a^{\Theta }$.

Akin to Eq. (13), site-fraction of a component in regular lattice of phase-$\Theta$ can be estimated from its respective mole-fraction through

14 $$\begin{aligned} y_{\mathrm{sub}:i}^{\Theta }=\frac{c_{\mathrm{sub}:i}^{\Theta }}{1-\sum _{k=1}^{l} c_{\mathrm{ints}:k}^{\Theta }}\left( = \frac{c_{\mathrm{sub}:i}^{\Theta }}{\sum _{i=1}^{j} c_{\mathrm{sub}:i}^{\Theta }} \right) . \end{aligned}$$

Evidently, as opposed to Eq. (13), the above conversion of mole- to site-fraction involving regular-lattice species is independent of the site ratio $a^{\Theta }$.

Even though Eqs. (13) and  (14) relate site- and mole-fraction of components in phase-$\Theta$, these expressions do not directly correspond to Eq. (9), wherein mole-fraction of an alloying element is calculated from its site-fraction. Nevertheless, a relation analogous to Eq. (9) can be derived, under the current consideration, by describing the mole-fraction of an interstitial component-*i* as

15 $$\begin{aligned} c_{\mathrm{ints}:k}^{\Theta } \left( = \frac{N_{\mathrm{ints}:k}^{\Theta }}{\sum _{k=1}^{l}N_{\mathrm{ints}:k}^{\Theta }+\sum _{i=1}^{j}N_{\mathrm{sub}:i}^{\Theta }}\right) = y_{\mathrm{ints}:k}^{\Theta }\frac{\sum _{k=1}^{l}N_{\mathrm{ints}:k}^{\Theta }+N_{\mathrm{ints}:va}^{\Theta }}{\sum _{k=1}^{l}N_{\mathrm{ints}:k}^{\Theta }+\sum _{i=1}^{j}N_{\mathrm{sub}:i}^{\Theta }}. \end{aligned}$$

By introducing the site-ratio constant $a^{\Theta }$ in Eq. (11), mole-fraction of interstitial components can be ascertained from site-fractions through

16 $$\begin{aligned} c_{\mathrm{ints}:k}^{\Theta } = y_{\mathrm{ints}:k}^{\Theta } \left[ \frac{a^{\Theta }}{1+a^{\Theta }\sum _{k=1}^{l}y_{\mathrm{ints}:k}^{\Theta }}\right] \left( =y_{\mathrm{ints}:k}^{\Theta } \left[ \frac{a^{\Theta }}{1+a^{\Theta }(1-y_{\mathrm{ints}:va}^{\Theta })}\right] \right) . \end{aligned}$$

Similarly, the relation

17 $$\begin{aligned} c_{\mathrm{sub}:i}^{\Theta } = y_{\mathrm{sub}:i}^{\Theta } \left[ \frac{1}{1+a^{\Theta }\sum _{k=1}^{l}y_{\mathrm{ints}:k}^{\Theta }}\right] \left( =y_{\mathrm{sub}:i}^{\Theta } \left[ \frac{1}{1+a^{\Theta }(1-y_{\mathrm{ints}:va}^{\Theta })}\right] \right) \end{aligned}$$

translates site-fraction of a regular-lattice species into mole-fraction. Unlike Eq. (14), the above expression which determines mole-fraction of a substitutionally-diffusing alloying element from its site-fractions is dependent on the site-ratio constant, $a^{\Theta }$.

### Site-fraction based grand-potential model

As opposed to the existing models^28–31^, in the present work, a site-fraction based phase-field technique is formulated in the grand-potential framework as a computationally efficient alternate for distinguishing interstitial and substitutional diffusion^19^. To that end, the characteristic aspects of the thermodynamically consistent two-phase approach, wherein the contribution of the bulk phases and interface are sufficiently decoupled, is considered as an appropriate starting point for the derivation.

In two-phase framework^24,25^, energy contribution of the bulk region constituting of phases-$\alpha$ and -$\beta$ is characteristically written as

18 $$\begin{aligned} f(\varvec{c}^{\alpha }, \varvec{c}^{\beta }, \phi ) = f^{\alpha }(\varvec{c}^{\alpha })h(\phi )+f^{\beta }(\varvec{c}^{\beta })(1-h(\phi )), \end{aligned}$$

where $\varvec{c}^{\alpha }$ and $\varvec{c}^{\beta }$ are, as delineated in Eq. (4), tuple of all independent phase-dependent concentrations represented in mole-fraction. In Eq. (18), the overall bulk contribution comprises of free-energy densities of individual phases, $f^{\alpha }(\varvec{c}^{\alpha })$ and $f^{\beta }(\varvec{c}^{\beta })$, interpolated through a smoothly varying monotonic function, which is of the form $h(\phi )=\phi ^2(3-2\phi )$, in the present formulation. Moreover, in the two-phase approach, the individual concentrations are interpolated into a continuous variable, $c_{i}$, as

19 $$\begin{aligned} c_{i}=c_{i}^{\alpha }h(\phi )+c_{i}^{\beta }(1-h(\phi )) \qquad \forall \; i\in \{1,\dots ,m\}, \end{aligned}$$

which facilitates in ascertaining previously unknown phase-dependent concentration under local-equilibrium assumption^25^. Even though a unique smooth function can be employed for interpolating concentration, given the rather simplistic consideration of the current derivation, and substantiated by outcomes of the previous investigations^33,34^, $h(\phi )$ involved in Eq. (18) is reasonably used for formulating continuous variable as well.

The energy contribution of the phases in Eq. (18) is augmented to the interface contribution, as described in Eq. (1), to yield an overall energy-density functional. The bulk driving-force that constitutes the phenomenological minimisation of the energy-density functional, and ultimately directs the spatio-temporal evolution of the phase-field, is expressed as

20 $$\begin{aligned} \frac{\delta {\mathcal {F}}_{\mathrm{bulk}}}{\delta \phi }&= \frac{\partial f(\varvec{c}^{\alpha }, \varvec{c}^{\beta }, \phi )}{\partial \phi } \\&= \left[ \Delta ^{\alpha }(\varvec{c}^{\alpha }) - \Delta ^{\beta }(\varvec{c}^{\beta }) \right] \frac{\partial h(\phi )}{\partial \phi }. \end{aligned}$$

The contribution of the individual phases to the bulk driving-force in above formulation reads

21 $$\begin{aligned} \Delta ^{\Theta }(\varvec{c}^{\Theta }) = f^{\Theta }(\varvec{c}^{\Theta }) - \sum _{i=2}^{m}\mu _{i}c_{i}^{\Theta }, \end{aligned}$$

where $$\Theta \in \{\alpha ,\beta \}$$^24^.

In Eq. (21), the number of concentration variables are reduced from *m* to $m-1$ through the summation relation associated with mole-fraction. Correspondingly, $\mu _{i}$ represents the diffusion potential of component-*i* with respect to matrix ($i=1$)^25^. In other words, while the $m-1$ independent concentrations are realised from characteristic mole-fraction relation ($\sum _{i=1}^{m}c_{i}^{\Theta }=1$), the number of its conjugate pair is appropriately reduced by considering diffusion potential ($\mu _{i}={\tilde{\mu }}_{i}-{\tilde{\mu }}_{1}$) instead of chemical potential, ${\tilde{\mu }}_{i}$. However, the incorporation of the mole-fraction based summation relation to identify the independent variables poses a critical hindrance in distinguishing components occupying interstitial and regular lattice^28^. Consequently, in the present derivation, the contribution of the individual phases to the bulk driving-force is re-formulated with *m* concentration variables as

22 $$\begin{aligned} \Delta ^{\Theta }(\varvec{c}^{\Theta }) = f^{\Theta }(\varvec{c}^{\Theta }) - \sum _{i=1}^{m}{\tilde{\mu }}_{i}c_{i}^{\Theta }, \end{aligned}$$

where ${\tilde{\mu }}_{i}$ is the chemical potential of species-*i*. Through appropriate conversion invoking relations analogous to Eqs. (13) and  (14), the free-energy density of the individual phases, and subsequently, the overall bulk contribution can be formulated based on site-fractions. The driving force resulting from the site-fraction based bulk contribution reads

23 $$\begin{aligned} \Delta ^{\Theta }(\varvec{y}^{\Theta }) = f^{\Theta }(\varvec{y}^{\Theta }) - \sum _{i=1}^{m}{\tilde{\mu }}_{i}y_{i}^{\Theta }, \end{aligned}$$

where tuple $\varvec{y}^{\Theta }$ includes concentration of all *m*-chemical species in site-fractions, and ${\tilde{\mu }}_{i}$ is the corresponding chemical potential of component-*i*.

In a multicomponent system, wherein the constituting elements occupy definite sublattices, the associated chemical potential adopts a ‘compound’ form^35^. Chemical potential of an element-*i*, in phases-$\Theta '$ with *s* sublattices, is written as

24 $$\begin{aligned} {\tilde{\mu }}_{i}^{\Theta '}= G_{m}^{\Theta '}+\sum _{\mathrm{s}}\frac{\partial G_{m}^{\Theta '}}{\partial y_{\mathrm{s}:i}}-\sum _{\mathrm{s}}\sum _{i=1}^{m+1}\frac{\partial G_{m}^{\Theta '}}{\partial y_{\mathrm{s}:i}}, \end{aligned}$$

where $G_{m}^{\Theta '}$ denotes the molar Gibbs free-energy density. When certain components in phase-$\Theta$ occupy interstitial sites, the corresponding chemical potential can be expressed based on the compound formalism described in Eq. (24).

By adopting the delineation in Eq. (10), *m*-components in phase-$\Theta$ are distinguished into regular-lattice and interstitial elements with *j*- and *l*(+1)-entities each, respectively. In this distinction, while the set of *j*-elements pertaining to the regular-lattice include matrix-species ($\mathrm{sub}:i=1$), vacancies associated with the interstitial site are not a part of the corresponding *l*-components.

Chemical potential of the alloying elements, and matrix-species, restricted to the regular-lattice sites, based on compound formulation in Eq. (24), is expressed as

25 $$\begin{aligned} {\tilde{\mu }}_{\mathrm{sub}:i}^{\Theta }=G_{m}^{\Theta } + \frac{\partial G^{\Theta }_{m}}{\partial y^{\Theta }_{\mathrm{sub}:i}} - \sum _{i=1}^{j} y^{\Theta }_{\mathrm{sub}:i} \frac{\partial G^{\Theta }_{m}}{\partial y^{\Theta }_{\mathrm{sub}:i}}, \end{aligned}$$

where site-fraction of substitutionally-diffusing component-*i* is represented by $y^{\Theta }_{\mathrm{sub}:i}$. Similarly, the respective chemical potential of species confined to the interstitial sites is of the form

26 $$\begin{aligned} {\tilde{\mu }}_{\mathrm{ints}:k}^{\Theta }=G_{m}^{\Theta } + \frac{\partial G^{\Theta }_{m}}{\partial y^{\Theta }_{\mathrm{ints}:k}} - \sum _{k=1}^{l+1} y^{\Theta }_{\mathrm{ints}:k} \frac{\partial G^{\Theta }_{m}}{\partial y^{\Theta }_{\mathrm{ints}:k}}, \end{aligned}$$

wherein $y^{\Theta }_{\mathrm{ints}:k}$ denotes the site-fraction of component-*k* in phase-$\Theta$. In Eq. (26), it should be noted that the consideration of ($l+1$)-entities facilities the inclusion of vacant sites in the formulation.

Expressing concentration in site-fraction lends itself to realising interstitial and regular-lattice components, and separately identifying the independent variables. For instance, since $\sum _{i=1}^{j} y^{\Theta }_{\mathrm{sub}:i}=1$, the evolution of the matrix-species can be known from the corresponding spatio-temporal changes exhibited by the alloying elements occupying the regular lattice. Similarly, interstitial diffusion of the associated components dictate the temporal evolution of vacancies. The conjugate pair for the independent concentrations are appropriately determined by considering the chemical potential of matrix-species and vacancy for regular and interstitial components, respectively. Accordingly, diffusion potential of regular-lattice component-*i*, written as

27 $$\begin{aligned} \mu _{\mathrm{sub}:i}^{\Theta } = {\tilde{\mu }}_{\mathrm{sub}:i}^{\Theta } - {\tilde{\mu }}_{\mathrm{sub}:1}^{\Theta } = \frac{\partial G^{\Theta }_{m}}{\partial y^{\Theta }_{\mathrm{sub}:i}} - \frac{\partial G^{\Theta }_{m}}{\partial y^{\Theta }_{\mathrm{sub}:1}}, \end{aligned}$$

conjugates the respective independent site-fraction based concentration. In Eq. (27), although the chemical potentials of alloying elements-*i*, ${\tilde{\mu }}_{\mathrm{sub}:i}^{\Theta }$, and matrix-species, ${\tilde{\mu }}_{\mathrm{sub}:1}^{\Theta }$, adopt a compound form expressed in Eq. (25), their difference by cancelling-out the individual energy-density, and other summation terms, yields a rather straightforward description of the diffusion potential.

Identical treatment extended to the interstitial components renders suitable conjugate pair for the respective independent concentrations. However, considering that the contribution of the interstitial vacancy to the corresponding chemical potential is relatively negligible, $\frac{\partial G^{\Theta }_{m}}{\partial y^{\Theta }_{\mathrm{ints}:va}}<<\frac{\partial G^{\Theta }_{m}}{\partial y^{\Theta }_{\mathrm{ints}:k}}$, the diffusion-potential of the component-*k* reads

28 $$\begin{aligned} \mu _{\mathrm{ints}:k}^{\Theta } = {\tilde{\mu }}_{\mathrm{ints}:k}^{\Theta } - {\tilde{\mu }}_{\mathrm{ints}:va}^{\Theta } = \frac{\partial G^{\Theta }_{m}}{\partial y^{\Theta }_{\mathrm{ints}:k}}. \end{aligned}$$

Comparing Eqs. (27) and  (28), it is evident that, while the diffusion potential of the regular-lattice element ($\mu _{\mathrm{sub}:i}^{\Theta }$) considers the difference in the chemical potentials, the corresponding conjugate pair of the interstitial component, $\mu _{\mathrm{ints}:k}^{\Theta }$, appears as a ‘regular‘ chemical potential. In other words, whereas the substitutional migration of the alloying elements is associated with the diffusion potential, ‘chemical potential’ of the individual component primarily governs its interstitial diffusion^36^.

Having distinguished the diffusion potentials of components occupying regular and interstitial site respectively through Eqs. (27) and  (28), the quasi-equilibrium condition, which is assumed across the diffuse interface, is separately written as

29 $$\begin{aligned} \mu _{\mathrm{sub}:i}^{\alpha }(\varvec{x},t)&=\mu _{\mathrm{sub}:i}^{\beta }(\varvec{x},t)\equiv \mu _{\mathrm{sub}:i}(\varvec{x},t) \qquad \forall \; i\in \{2,\dots ,j\} \\ \mu _{\mathrm{ints}:k}^{\alpha }(\varvec{x},t)&=\mu _{\mathrm{ints}:k}^{\beta }(\varvec{x},t)\equiv \mu _{\mathrm{ints}:k}(\varvec{x},t) \qquad \forall \; k\in \{1,\dots ,l\}. \end{aligned}$$

Correspondingly, the bulk driving-force of a system with regular and interstitial lattice, in this framework, is expressed as

30 $$\begin{aligned} \Delta ^{\Theta }(\varvec{y}_{\mathrm{sub}}^{\Theta },\varvec{y}_{\mathrm{ints}}^{\Theta }) = f^{\Theta }(\varvec{y}_{\mathrm{sub}}^{\Theta },\varvec{y}_{\mathrm{ints}}^{\Theta }) - \sum _{i=2}^{j}\mu _{\mathrm{sub}:i}y_{\mathrm{sub}:i}^{\Theta } - \sum _{k=1}^{l}\mu _{\mathrm{ints}:k}y_{\mathrm{ints}:k}^{\Theta }, \end{aligned}$$

where the number of independent variables amounts to $m-1$. Despite distinguishing the components of the system based on their lattice position, the fictitious phase-dependent concentrations continue to remain the fundamental variable of the bulk driving-force, in Eq. (30), characterising the two-phase approach. A change in the fundamental variable and a consequent shift to the grand-potential technique, all-while retaining the distinctive features of the current site-fraction based formulation, is established by considering the Legendre transform of the individual free-energy densities.

The Legendre transform of the site-fraction based energy-densities of the individual phases yields corresponding grand chemical-potential, which is expressed as

31 $$\begin{aligned} \psi ^{\Theta }(\varvec{y}^{\Theta }(\varvec{\mu }_{\mathrm{sub}},\varvec{\mu }_{\mathrm{ints}})) = f^{\Theta }(\varvec{y}^{\Theta }(\varvec{\mu }_{\mathrm{sub}},\varvec{\mu }_{\mathrm{ints}})) - \sum _{i=2}^{j}\mu _{\mathrm{sub}:i}y_{\mathrm{sub}:i}^{\Theta }(\mu _{\mathrm{sub}:i}) - \sum _{k=1}^{l}\mu _{\mathrm{ints}:k}y_{\mathrm{ints}:k}^{\Theta }(\mu _{\mathrm{ints}:k}), \end{aligned}$$

where $\varvec{y}^{\Theta }$ reflects the delineation in Eq. (10). The fundamental variables of the grand chemical-potential, owing to the distinction in components occupying regular and interstitial lattice, are different from the existing representation in Eq. (7), and are written as

32 $$\begin{aligned} \varvec{\mu }_{\mathrm{sub}}&=\{\mu _{\mathrm{sub}:2}, \mu _{\mathrm{sub}:3},\dots ,\mu _{\mathrm{sub}:j}\} \\ \varvec{\mu }_{\mathrm{ints}}&=\{\mu _{\mathrm{ints}:1}, \mu _{\mathrm{ints}:2},\dots ,\mu _{\mathrm{ints}:l}\}, \end{aligned}$$

where each entries, depending on the lattice position of the component, adhere to the description rendered by Eqs. (27) or  (28). When viewed in relation to Eq. (30), the grand potential in Eq. (31) is of the form similar to individual bulk driving-force. Therefore, the bulk driving-force in the grand-potential formalism is the difference in the individual potential-densities, $\psi ^{\alpha }(\varvec{y}^{\alpha }(\varvec{\mu }_{\mathrm{sub}},\varvec{\mu }_{\mathrm{ints}})) - \psi ^{\beta }(\varvec{y}^{\beta }(\varvec{\mu }_{\mathrm{sub}},\varvec{\mu }_{\mathrm{ints}}))$.

Similar to the two-phase approach, the overall energy-density of the system in the grand-potential framework is formulated by interpolating the energy-density contribution of the individual phases. Accordingly, the grand chemical-potential of the entire system is expressed as

33 $$\begin{aligned} \psi (\varvec{\mu }_{\mathrm{sub}},\varvec{\mu }_{\mathrm{ints}}) = \psi ^{\alpha }(\varvec{y}^{\alpha }(\varvec{\mu }_{\mathrm{sub}},\varvec{\mu }_{\mathrm{ints}})) h(\phi ) + \psi ^{\beta }(\varvec{y}^{\beta }(\varvec{\mu }_{\mathrm{sub}},\varvec{\mu }_{\mathrm{ints}})) (1-h(\phi )), \end{aligned}$$

where $\psi ^{\alpha }$ and $\psi ^{\beta }$ are individual grand potential-densities of phase-$\alpha$ and -$\beta$, respectively. The interpolation function introduced earlier, $h(\phi )=\phi ^2(3-2\phi )$, is employed in the current grand potential formulation.

In the two-phase approach, the previously unknown phase-dependent concentrations are estimated from the local-equilibrium conditions, and the description of the continuous concentration. However, following existing framework of the grand-potential formalism^19,20^, the fictitious concentrations are ascertained by considering the invertibility of the continuous fundamental variable. Accordingly, the following relation

34 $$\begin{aligned} \frac{\partial \psi ^{\alpha }(\varvec{y}^{\alpha }(\varvec{\mu }_{\mathrm{sub}},\varvec{\mu }_{\mathrm{ints}}))}{\partial \mu _{\mathrm{sub}:i}} = -y_{\mathrm{sub}:i}^{\alpha } \end{aligned}$$

can be employed to determine the concentration of regular-lattice component-*i* in phase-$\Theta$. From Eqs. (33) and  (34), an expression for continuous concentration as in Eq. (19) can be recovered. However, owing to the distinct consideration of the substitutionally and interstitially diffusing components, the continuous concentrations are separately expressed as

35 $$\begin{aligned} y_{\mathrm{sub}:i}&= y_{\mathrm{sub}:i}^{\alpha }h(\phi ) + y_{\mathrm{sub}:i}^{\beta }(1-h(\phi )) \\ y_{\mathrm{ints}:k}&= y_{\mathrm{ints}:k}^{\alpha }h(\phi ) + y_{\mathrm{ints}:k}^{\beta }(1-h(\phi )), \end{aligned}$$

In the present formulation, a monotonic function of identical form is adopted for interpolating grand-potential densities, and site-fraction based concentration of regular-lattice and interstitial chemical species.

### Governing evolution equations

Having introduced site-fraction based grand chemical-potential by starting from the principal considerations of two-phase approach, the corresponding energy-density functional is formulated by including the contributions of the diffuse interface. This grand-potential functional for a system with two phases, and volume *V*, is written as

36 $$\begin{aligned} \Omega (\varvec{\mu }_{\mathrm{sub}},\varvec{\mu }_{\mathrm{ints}}, \phi , \varvec{\nabla }\phi )=\int _{V}\mathrm{d}{V} \left[ \psi (\varvec{\mu }_{\mathrm{sub}},\varvec{\mu }_{\mathrm{ints}}, \phi ) + \frac{1}{\epsilon } f_{\mathrm{pen}}(\phi ) + \epsilon \gamma | \varvec{\nabla }\phi |^{2} \right] , \end{aligned}$$

where diffusion and chemical potentials of components occupying regular and interstitial lattice, $\varvec{\mu }_{\mathrm{sub}}$ and $\varvec{\mu }_{\mathrm{ints}}$, act as the fundamental dynamic variables^36^. In Eq. (36), the interface contribution comprises of penalising potential-density represented by $\frac{1}{\epsilon } f_{\mathrm{pen}}(\phi )$ and gradient energy density, $\epsilon \gamma | \varvec{\nabla }\phi |^{2}$. The parameter $\epsilon$ associated with the penalising- and gradient-potential is a length-scale factor, which governs the width of the diffuse interface, and $\gamma$, in Eq. (36), is the interfacial energy-density. In the present formulation, a rather straightforward double-well potential, $f_{\mathrm{pen}}(\phi )=\frac{16}{\pi ^2}\gamma \phi ^2(1-\phi )^2$, is employed to penalise phase-field, and ensure that a constant value ($\phi =1$, 0) is assumed at the either ends of the diffuse-interface region^20^.

The spatio-temporal changes in phase-field, which translates into the perceivable transformation in the simulation domain, is devised by considering a phenomenological reduction of the overall energy-density functional described in Eq. (36). Correspondingly, the evolution equation of phase-field reads

37 $$\begin{aligned} \tau \epsilon \frac{\partial \phi }{\partial t}&= -\frac{\delta \Omega (\varvec{\mu }_{\mathrm{sub}},\varvec{\mu }_{\mathrm{ints}}, \phi , \varvec{\nabla }\phi )}{\delta \phi } \\&= 2\gamma \epsilon \varvec{\nabla }^{2}\phi - \frac{1}{\epsilon }\frac{\partial f_{\mathrm{pen}}(\phi )}{\partial \phi } - \underbrace{\left[ \psi ^{\alpha }(\varvec{y}^{\alpha }(\varvec{\mu }_{\mathrm{sub}},\varvec{\mu }_{\mathrm{ints}})) - \psi ^{\beta }(\varvec{y}^{\beta }(\varvec{\mu }_{\mathrm{sub}},\varvec{\mu }_{\mathrm{ints}})) \right] }_{\Delta \psi (\varvec{\mu }_{\mathrm{sub}},\varvec{\mu }_{\mathrm{ints}})} \frac{\partial h(\phi )}{\partial \phi }, \end{aligned}$$

where $\tau$ is the relaxation parameter that ensures the stability of the interface during its migration. As discussed earlier, the bulk driving-force governing the spatio-temporal evolution of phase-field is the difference in the grand-potential densities of the individual phases, $\Delta \psi (\varvec{\mu }_{\mathrm{sub}},\varvec{\mu }_{\mathrm{ints}})=\psi ^{\alpha }(\varvec{\mu }_{\mathrm{sub}},\varvec{\mu }_{\mathrm{ints}})-\psi ^{\beta }(\varvec{\mu }_{\mathrm{sub}},\varvec{\mu }_{\mathrm{ints}})$.

Since the bulk driving-force, $\Delta \psi (\varvec{\mu }_{\mathrm{sub}},\varvec{\mu }_{\mathrm{ints}})$, is primarily dictated by the diffusion and chemical potential of regular and interstitial alloying elements, the temporal evolution of these dynamic variable, in principle, govern the changes in phase-field. By adhering the existing grand-potential framework^19,26^, the temporal evolution of the dynamic variables are formulated as

38 $$\begin{aligned} \frac{\partial \mu _{i}}{\partial t} = \Bigg [ \underbrace{\varvec{\chi }^{\alpha }_{ik}h(\phi ) + \varvec{\chi }^{\beta }_{ik}(1-h(\phi ))}_{:=\varvec{\chi }(\phi )} \Bigg ]^{-1} \Bigg [ \underbrace{\varvec{\nabla }\cdot \sum _{i\le k}^{m-1} \varvec{M}_{ik}(\phi ) \varvec{\nabla }\mu _{k}}_{:=\frac{\partial y_{i}}{\partial t}} - (y^{\alpha }_{i} - y^{\beta }_{i}) \frac{\partial h(\phi )}{\partial t} \Bigg ], \end{aligned}$$

where $\varvec{\chi }^{\Theta }_{ik}$ is the susceptibility matrix, wherein each entries are the inverse of the second-derivative of free-energy densities with respect to appropriate concentrations. The mobility parameter, $\varvec{M}_{ik}(\phi )$, governing the rate of evolution of the fundamental variable is expressed as

39 $$\begin{aligned} \varvec{M}_{ik}(\phi )=\varvec{D}^{\alpha }_{ik}\varvec{\chi }^{\alpha }_{ik}h(\phi ) + \varvec{D}^{\beta }_{ik}\varvec{\chi }^{\beta }_{ik}(1-h(\phi )), \end{aligned}$$

where $\varvec{D}^{\Theta }_{ik}$ is the interdiffusivity matrix identical in form and dimension to the corresponding susceptibility matrix. The monotonic function employed to interpolate energy-densities of the individual phases is involved in the formulation of mobility in Eq. (39). Despite the differences in the fundamental-dynamic variables of interstitial and regular-lattice components, by appropriately devising the individual terms of the evolution equation, including mobility and susceptibility matrix^20^, the temporal changes in the diffusion (or chemical) potential can be expressed in an unified manner, as in Eq. (38). Moreover, for the present system, mobility and susceptibility matrices are of identical dimension ($m-1 \times m-1$).

## Energy density description

As elucidated earlier, the evolution of phase-field is largely dictated by the driving-force introduced by the bulk contribution. Irrespective of the theoretical framework, a physical transformation can only be modelled when a quantitative driving-force is introduced in the formulation. Therefore, by appropriately describing the thermodynamic parameters, like free-energy densities and diffusion (or chemical) potential, based on established CALPHAD databases^37^, the microstructural evolutions can be quantitatively modelled in phase-field technique^34,38^.

### Free-energy approximation

In order to render quantitative driving-force, by incorporating CALPHAD-informed thermodynamic data, different approaches have been adopted in phase-field formulation^39,40^. Amongst others, one of the commonly employed method involves accessing, and introducing, the CALPHAD data parallely while solving the evolution of phase-field and independent dynamic-variables^41^. Alternatively, free-energy densities of the individual phases have been numerically approximated based on the established databases^42^. Owing to its computational efficiency, and proven ability to quantitatively model microstructural evolution^33,43,44^, this ‘fitting’ approach is extended for site-fraction based formulation, wherein the system considered comprises of phases with interstitial components.

Extending the existing isothermal formulation of parabolic approximation^19^, site-fraction based free-energy density of individual phases with alloying elements occupying interstitial sites can be expressed as

40 $$\begin{aligned} f^{\Theta }(\varvec{y}^{\Theta }_{\mathrm{sub}},\varvec{y}^{\Theta }_{\mathrm{ints}}) = \frac{1}{V_m}\left[ \sum _{i=2}^{j}{\mathcal {A}}^{\Theta }_{\mathrm{sub}:i}\left( y^{\Theta }_{\mathrm{sub}:i} - y^{\Theta }_{\mathrm{sub}:i_{eq}} \right) ^2 + \sum _{k=1}^{l}{\mathcal {A}}^{\Theta }_{\mathrm{ints}:k}\left( y^{\Theta }_{\mathrm{ints}:k} - y^{\Theta }_{\mathrm{ints}:k_{eq}} \right) ^2 \right] , \end{aligned}$$

where $y^{\Theta }_{\mathrm{sub}:i_{eq}}$ and $y^{\Theta }_{\mathrm{ints}:k_{eq}}$ correspond to the equilibrium concentration, in site-fraction, of regular-lattice and interstitial alloying elements *i* and *k*. In Eq. (40), the identical molar volume of the phases are represented by $V_m$. The coefficients ${\mathcal {A}}^{\Theta }_{\mathrm{sub}:i}$ and ${\mathcal {A}}^{\Theta }_{\mathrm{ints}:k}$ are appropriately ascertained from the CALPHAD database in order to facilitate the recovery of thermodynamic parameters, including diffusion (or chemical) potential.

From the free-energy approximation in Eq. (40), the diffusion potential of the regular-lattice component-*i* is written as

41 $$\begin{aligned} \mu _{\mathrm{sub}:i} = \frac{1}{V_m}\left[ 2{\mathcal {A}}^{\Theta }_{\mathrm{sub}:i}\left( y^{\Theta }_{\mathrm{sub}:i} - y^{\Theta }_{\mathrm{sub}:i_{eq}} \right) \right] . \end{aligned}$$

Similarly, the ‘chemical’ potential of the interstitial alloying element-*k* reads

42 $$\begin{aligned} \mu _{\mathrm{ints}:k} = \frac{1}{V_m}\left[ 2{\mathcal {A}}^{\Theta }_{\mathrm{ints}:k}\left( y^{\Theta }_{\mathrm{ints}:k} - y^{\Theta }_{\mathrm{ints}:k_{eq}} \right) \right] . \end{aligned}$$

Given the invertibility of the diffusion (or chemical) potential, the previously unknown fictitious phase-dependent concentration, which aides in decoupling the bulk and interface energy contribution, can be determined from Eqs. (41) and  (42). Accordingly, phase-dependent concentration of components-*i* and -*k* associated with regular and interstitial lattice, in site-fraction, is estimated by

43 $$\begin{aligned} y^{\Theta }_{\mathrm{sub}:i}(\mu _{\mathrm{sub}:i})&= y^{\Theta }_{\mathrm{sub}:i_{eq}} + \mu _{\mathrm{sub}:i}\left( \frac{V_m}{2{\mathcal {A}}^{\Theta }_{\mathrm{sub}:i}} \right) \\ y^{\Theta }_{\mathrm{ints}:k}(\mu _{\mathrm{ints}:k})&= y^{\Theta }_{\mathrm{ints}:k_{eq}} + \mu _{\mathrm{ints}:k}\left( \frac{V_m}{2{\mathcal {A}}^{\Theta }_{\mathrm{ints}:k}} \right) . \end{aligned}$$

In the above expression, it is critical to note that the equilibrium concentrations of the components, irrespective of their lattice positions, are constant like the coefficients, ${\mathcal {A}}^{\Theta }_{\mathrm{sub}:i}$ and ${\mathcal {A}}^{\Theta }_{\mathrm{ints}:k}$, for a given phase at a specific temperature. By substituting Eq. (43) in Eq. (40), the free-energy density of the individual phases can be written as

44 $$\begin{aligned} f^{\Theta }(\varvec{y}^{\Theta }_{\mathrm{sub}}(\varvec{\mu }_{\mathrm{sub}}),\varvec{y}^{\Theta }_{\mathrm{ints}}(\varvec{\mu }_{\mathrm{ints}})) = \frac{V_m}{4}\left[ \sum _{i=2}^{j} \left( \frac{1}{{\mathcal {A}}^{\Theta }_{\mathrm{sub}:i}} \right) \mu _{\mathrm{sub}:i}^{2} + \sum _{k=1}^{l} \left( \frac{1}{{\mathcal {A}}^{\Theta }_{\mathrm{ints}:k}} \right) \mu _{\mathrm{ints}:k}^{2} \right] . \end{aligned}$$

The above description of the free-energy density, which forms the basis for the present formulation, is principally governed by the continuous variable, $\varvec{\mu }_{\mathrm{sub}}$ and $\varvec{\mu }_{\mathrm{ints}}$, representing diffusion and chemical potential of components in regular and interstitial lattice, respectively.

### Resulting driving-force

By substituting the free-energy density, Eq. (44), along with the potential based description of phase-dependent concentrations, Eq. (43), in Eq. (31), the grand chemical-potential of individual phases is re-formulated as

45 $$\begin{aligned} \psi ^{\Theta }(\varvec{\mu }_{\mathrm{sub}},\varvec{\mu }_{\mathrm{ints}}) = -\frac{V_m}{2}\left\{ \sum _{i=2}^j \left[ \left( \frac{1}{{\mathcal {A}}^{\Theta }_{\mathrm{sub}:i}} \right) \mu _{\mathrm{sub}:i}^{2} + \mu _{\mathrm{sub}:i} y^{\Theta }_{\mathrm{sub}:i_{eq}} \right] + \sum _{k=1}^l \left[ \left( \frac{1}{{\mathcal {A}}^{\Theta }_{\mathrm{ints}:k}} \right) \mu _{\mathrm{ints}:k}^{2} + \mu _{\mathrm{ints}:k} y^{\Theta }_{\mathrm{ints}:k_{eq}} \right] \right\} . \end{aligned}$$

Introducing the above grand-potential density, which emerges from the second-order approximation of free-energy, in Eq. (36), a functional informing the overall energy of the system can be expressed. This functional during the phase-field evolution (Eq. (37)) yields a bulk driving-force of the form

46 $$\begin{aligned}&\Delta \psi (\varvec{\mu }_{\mathrm{sub}},\varvec{\mu }_{\mathrm{ints}}) = \psi ^{\alpha }(\varvec{\mu }_{\mathrm{sub}},\varvec{\mu }_{\mathrm{ints}}) - \psi ^{\beta }(\varvec{\mu }_{\mathrm{sub}},\varvec{\mu }_{\mathrm{ints}}) \\&= \frac{V_m}{2} \left( \sum _{i=2}^{j}\Delta \chi _{\mathrm{sub}:i}\mu _{\mathrm{sub}:i}^2 + \sum _{k=1}^{l}\Delta \chi _{\mathrm{ints}:k}\mu _{\mathrm{ints}:k}^2 \right) + \sum _{i=2}^{j}\Delta y_{\mathrm{sub}:i}\mu _{\mathrm{sub}:i} + \sum _{k=1}^{l}\Delta y_{\mathrm{ints}:k}\mu _{\mathrm{ints}:k}, \end{aligned}$$

where the associated terms are

47 $$\begin{aligned} \Delta \chi _{\mathrm{sub}:i} = \frac{1}{{\mathcal {A}}^{\beta }_{\mathrm{sub}:i}}-\frac{1}{{\mathcal {A}}^{\alpha }_{\mathrm{sub}:i}} \qquad&\mathrm{and} \qquad \Delta \chi _{\mathrm{ints}:k} = \frac{1}{{\mathcal {A}}^{\beta }_{\mathrm{ints}:k}}-\frac{1}{{\mathcal {A}}^{\alpha }_{\mathrm{ints}:k}} \\ \Delta y_{\mathrm{sub}:i} = y^{\beta }_{\mathrm{sub}:i_{eq}} - y^{\alpha }_{\mathrm{sub}:i_{eq}} \qquad&\mathrm{and} \qquad \Delta y_{\mathrm{ints}:k} = y^{\beta }_{\mathrm{ints}:k_{eq}} - y^{\alpha }_{\mathrm{ints}:k_{eq}}. \end{aligned}$$

Owing to the parabolic approximation of free-energy density, the individual entities of the susceptibility matrix are the reciprocal of the coefficients, ${\mathcal {A}}^{\Theta }_{\mathrm{sub}:i}$ or ${\mathcal {A}}^{\Theta }_{\mathrm{ints}:k}$^20^. Moreover, for a given composition, since equilibrium concentrations of phases vary only with temperature, $\Delta y_{\mathrm{sub}:i}$ and $\Delta y_{\mathrm{ints}:k}$ are constant under isothermal conditions. When the CALPHAD data is incorporated by numerically fitting the free-energy densities, Eq. (46) delineates the resulting bulk driving-force that dictates the temporal evolution of phase-field.

### Approximation encompassing coupling

Even though free-energy formulation in Eq. (40) appears to be a reasonable extension of the existing description for binary systems^45^, there is a noticeable limitation associated with it. Considering that the entries of the susceptibility matrix, under second-order approximation of energy-densities, are the inverse of coefficients (${\mathcal {A}}^{\Theta }_{\mathrm{sub}:i}$ or ${\mathcal {A}}^{\Theta }_{\mathrm{ints}:k}$), the extended formulation in Eq. (40) yields a diagonal matrix. When this form of susceptibility matrix, wherein non-diagonal entries are zero, is adopted to describe phase-field mobility, $\varvec{M}_{ik}(\phi )$ in Eq. (39), the influence of interdiffusivity gets significantly compromised. Therefore, a more suitable approximation of free-energy density for a multicomponent phase with alloying elements occupying interstitial sites is formulated as

48 $$\begin{aligned} f^{\Theta }(\varvec{y}^{\Theta }_{\mathrm{sub}},\varvec{y}^{\Theta }_{\mathrm{ints}}) = \frac{1}{V_m} \left[ \sum _{i\le k}^{m-1,m-1} {\mathcal {A}}^{\Theta }_{ik} \left( y^{\Theta }_{\mathrm{sub}:i} - y^{\Theta }_{\mathrm{sub}:i_{eq}} \right) \left( y^{\Theta }_{\mathrm{ints}:k} - y^{\Theta }_{\mathrm{ints}:k_{eq}} \right) \right] . \end{aligned}$$

Under the above description, since the non-diagonal entities of the susceptibility tensor are non-trivial, role of interdiffusivity is sustained without any compromise.

By separating the coefficients corresponding to the diagonal entries, the free-energy density of phase-$\Theta$ can be written as

49 $$\begin{aligned}&f^{\Theta }(\varvec{y}^{\Theta }_{\mathrm{sub}},\varvec{y}^{\Theta }_{\mathrm{ints}}) = \frac{1}{V_m} \left[ \sum _{i=2}^{j}{\mathcal {A}}^{\Theta }_{ii} \left( y^{\Theta }_{\mathrm{sub}:i} - y^{\Theta }_{\mathrm{sub}:i_{eq}} \right) ^2 + \sum _{k=1}^{l} {\mathcal {A}}^{\Theta }_{kk}\left( y^{\Theta }_{\mathrm{ints}:k} - y^{\Theta }_{\mathrm{ints}:k_{eq}} \right) ^2 \right. \\&\quad \left. + \sum _{i<k}^{m-1,m-1} {\mathcal {A}}^{\Theta }_{ik} \left( y^{\Theta }_{\mathrm{sub}:i} - y^{\Theta }_{\mathrm{sub}:i_{eq}} \right) \left( y^{\Theta }_{\mathrm{ints}:k} - y^{\Theta }_{\mathrm{ints}:k_{eq}} \right) \right] . \end{aligned}$$

Diffusion potential of the chemical species occupying regular lattice, based above Eq. (49), reads

50 $$\begin{aligned} \mu _{\mathrm{sub}:i} = \frac{1}{V_m}\left[ 2{\mathcal {A}}^{\Theta }_{ii}\left( y^{\Theta }_{\mathrm{sub}:i} - y^{\Theta }_{\mathrm{sub}:i_{eq}} \right) + \sum _{k=1}^{l} {\mathcal {A}}^{\Theta }_{ik} \left( y^{\Theta }_{\mathrm{ints}:k} - y^{\Theta }_{\mathrm{ints}:k_{eq}} \right) \right] . \end{aligned}$$

Similarly, in the current formulation of the free-energy density, the chemical potential of the interstitial components adopts the form

51 $$\begin{aligned} \mu _{\mathrm{ints}:k} = \frac{1}{V_m}\left[ 2{\mathcal {A}}^{\Theta }_{kk}\left( y^{\Theta }_{\mathrm{ints}:k} - y^{\Theta }_{\mathrm{ints}:k_{eq}} \right) + \sum _{i=2}^{j} {\mathcal {A}}^{\Theta }_{ki} \left( y^{\Theta }_{\mathrm{sub}:i} - y^{\Theta }_{\mathrm{sub}:i_{eq}} \right) \right] . \end{aligned}$$

Ascertaining the previously unknown phase-dependent concentration from the free-energy approximation in Eq. (49) is not as straightforward, when compared to the corresponding description in Eq. (40). However, the formulation in Eq. (49) renders adequate number of relations to determine the unknown variables, *i*.*e* phase-dependent concentrations^46,47^. Despite the limitation, a discussion based on the free-energy approximation associated with Eq. (40) is offered in previous section solely to unravel the representative form of the bulk driving-force.

## Preliminary investigations

### Capturing sublattice stoichiometry

In a phase with sublattices, a stoichiometric relation might establish between the components depending on their lattice positions^48^. Phases with such relations are represented in a unique manner wherein the components of a sublattice are categorised together with a suffix depicting its stoichiometry. For instance, a hypothetical phase with three sublattices comprising of fictitious components; A, B and C, exhibiting stoichiometry, is expressed as (A,B)$_{{\tilde{h}}}$(B,C)$_{{\tilde{k}}}$(C,A)$_{{\tilde{l}}}$, where the species of the each sublattices are gathered separately, and the stoichiometry is represented by parameters ${\tilde{h}}$, ${\tilde{k}}$ and ${\tilde{l}}$. The stoichiometric parameters generally fulfill the condition ${\tilde{h}}+{\tilde{k}}+{\tilde{l}}=1$^31^.

**Figure 1:**
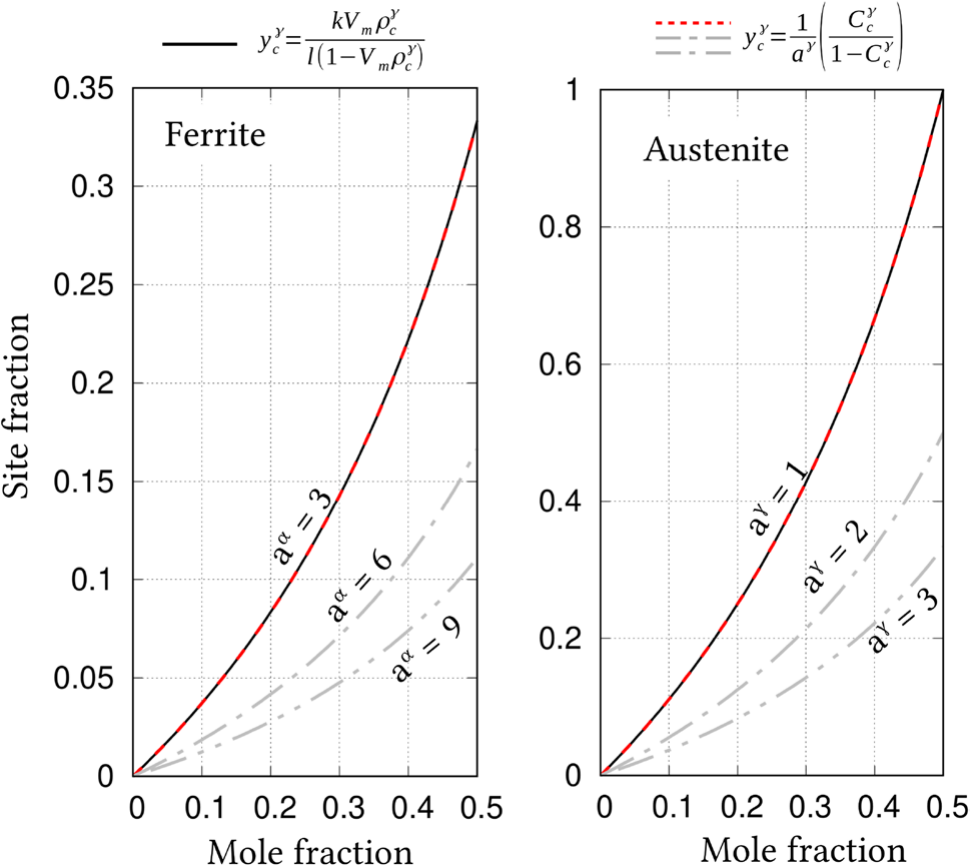
Relation between site- and mole-fraction, in ferrite and austenite, estimated based on Eqs. (13) and  (52). Constant, $a^{\Theta }$, involved in Eq. (13), varies with the *permissible interstitial-sites consideration*. When carbon atoms are assumed to occupy octahedral, tetrahedral and all sites of interstitial lattice, in ferrite, the constant correspondingly adopts the value of $a^{\Theta }=3$, 6 and 9, on the other hand, in austenite, it changes to $a^{\Theta }=1$, 2 and 3, respectively. In both ferrite and austenite, sublattice stoichiometry is recovered when the permissible interstitial-sites are confined to octahedral positions.

The stoichiometry associated with sublattices of a phase significantly influences the conversion of mole- to site-fraction. Since the conversion of concentrations plays a critical role in the present formulation, the ability of relations in Eqs. (13) and  (14) to recover the sublattice stoichiometry is verified. To that end, the phases involved in the subsequent discussions, *i*.*e* ferrite and austenite, are considered for the present investigation.

In its sublattice form, austenite and ferrite are correspondingly represented as (Fe)$_{0.5}$(C,Va)$_{0.5}$ and (Fe)$_{0.25}$(C,Va)$_{0.75}$, where carbon and vacancy associated with the interstitial sites are distinguished from the regular lattice exclusively occupied by iron^48^. For phases of this form, site-fractions of components confined to the interstitial sublattice are determined by

52 $$\begin{aligned} y_{C}^{\Theta }&= \frac{{\tilde{k}}V_m\rho _{C}^{\Theta }}{{\tilde{l}}(1-V_m\rho _{C}^{\Theta })}\left( =\frac{{\tilde{k}}c_{C}^{\Theta }}{{\tilde{l}}(1-c_{C}^{\Theta })}\right) \\ y_{Va}^{\Theta }&= \frac{{\tilde{l}}-V_m\rho _{C}^{\Theta }}{{\tilde{l}}(1-V_m\rho _{C}^{\Theta })}\left( =\frac{{\tilde{l}}-c_{C}^{\Theta }}{{\tilde{l}}(1-c_{C}^{\Theta })}\right) , \end{aligned}$$

where $\Theta$ represents austenite or ferrite ($\Theta \in \{\alpha , \gamma \}$), and $\rho _{C}^{\Theta }$ is the molar number-density of carbon^31^. The stoichiometric parameters ${\tilde{k}}$ and ${\tilde{l}}$ vary with phase. While ${\tilde{k}}={\tilde{l}}=0.5$ in austenite, ferrite is characterised by ${\tilde{k}}=0.25$ and ${\tilde{l}}=0.75$.

As opposed to Eq. (52), in the present approach, the site-fraction of a component is estimated using the lattice constant, $a^{\Theta }$, through Eqs. (13) and (14). In ferrite and austenite, the interstitial lattice comprises of octahedral and tetrahedral sites. Depending on the sites considered, the value of $a^{\Theta }$ varies. In Fig. 1, the relation between mole- and site-fraction, in both austenite and ferrite, for different lattice constants, $a^{\Theta }$, is graphically represented. The lattice constant assumes the value 3, 6 and 9, in ferrite, when carbon respectively occupies octahedral, tetrahedral and all interstitial sites. In contrast, under this *permissible interstitial-sites consideration*, carbon occupying octahedral and tetrahedral sites of austenite correspondingly renders the value of $a^{\gamma }$= 1 and 2.

A curve depicting Eq. (52), wherein the influence stoichiometric parameters are considered, is included in illustration Fig. 1  to explicate the ability of Eqs. (13) and (14) to recover the sublattice stoichiometry. Evidently, a plausible consideration that the carbon exclusively occupies octahedral sites of the interstitial lattice, $a^{\alpha }$=3 and $a^{\gamma }$=1, lends itself to the recovery sublattice stoichiometry in both ferrite and austenite. Therefore, as indicated by Fig. 1, it can be stated that the sublattice stoichiometry of any phases can be recovered in Eqs. (13) and (14) by appropriately devising the lattice constant, $a^{\Theta }$.

### Mole-fraction error

Concentration of chemical species constituting a multicomponent system can be expressed in various forms, including site- and mole-fractions. Despite the varied representations, when the system is static, *i*.*e* does not evolve with time, its chemical composition can be comprehended without the loss of any generality. However, in a dynamic system that evolves with time, it has been shown that a difference in the treatment of composition introduces noticeable disparity in the concentration distribution^28^. In other words, a rather inappropriate representation of concentration introduces a perceivable degree of inaccuracy in the evolution of components. Since the present work primarily aims to enhance the existing phase-field models by proper handling of composition, the diffusion of carbon accompanying the carburisation is modelled in a one-dimensional setup to emphasise the importance of accurate representation of concentration. Even though this adjunct study does not directly contribute to the phase-field approach, it amplifies the significance of employing site-fraction to delineate the composition of systems with interstitial sublattice.

**Figure 2:**
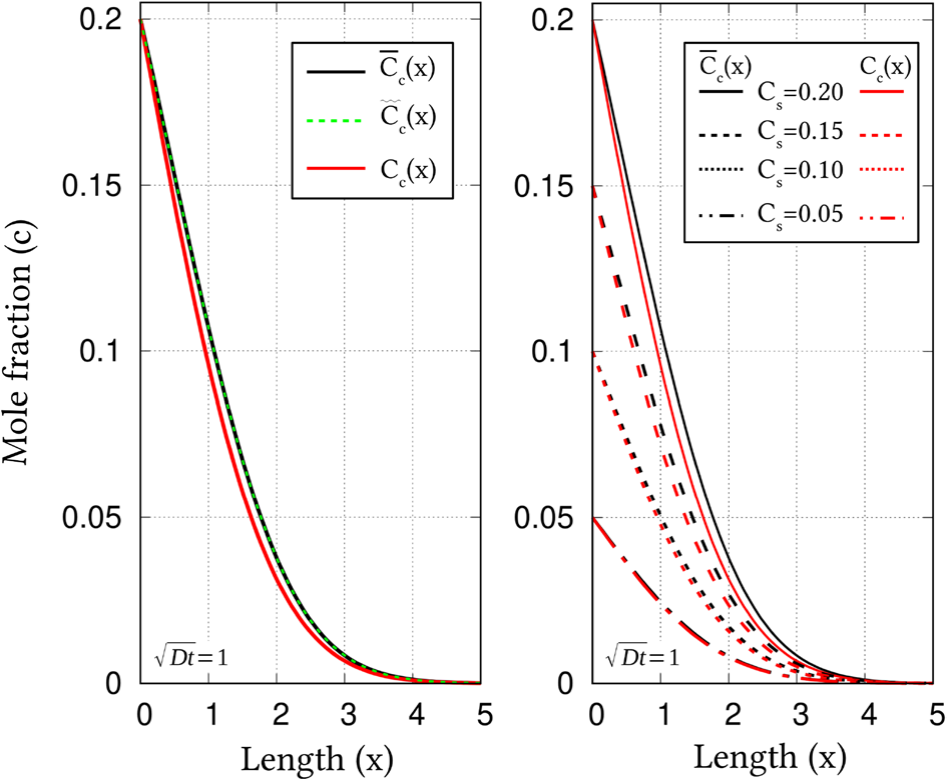
Concentration profile of carbon, at $\sqrt{Dt}=1$, reflecting the diffusion governed by mole- and site-fraction based formulations, respectively expressed in Eqs. (53) and  (55). On the left plot, wherein single surface-concentration is treated, concentration profile for a different ratio-constant, $a^{\Theta }$, is included. Concentration profile emerging from various surface-contents is graphically represented on the right.

Consider a one-dimensional system of binary ferrite with negligible carbon concentration in bulk, and relatively high carbon content in its surface. Owing to the significant gradient in the concentration, carbon from the surface diffuses into the ferrite matrix. When carbon content is treated in mole-fraction, the concentration evolution is dictated by

53 $$\begin{aligned} \frac{\partial c_C}{\partial t}= \frac{\partial }{\partial x}D_c\frac{\partial c_C}{\partial x}, \end{aligned}$$

where $D_c$ is the diffusivity of carbon. Solving the above concentration-evolution equation analytically, yields a solution

54 $$\begin{aligned} c_C(x,t)=c_{s}-c_{s}\mathrm{erf}\left( \frac{x}{2\sqrt{D_c t}}\right) , \end{aligned}$$

where $c_{s}$ is surface concentration of carbon expressed in mole-fraction. The error-function solution in Eq. (54) can be used to construct the one-dimensional concentration profile at any given time, *t*. Since carbon is represented in mole-fraction, the spatio-temporal evolution of matrix iron is ascertained by $c_{\mathrm{Fe}}(x,t) = 1 - c_C(x,t)$. However, such estimation assumes that there is a reverse diffusion of iron complementing the migration of carbon, which is rather inaccurate, particularly, given that carbon occupies the interstitial sites. Therefore, a more appropriate representation of carbon concentration would involve site-fraction. Correspondingly, the expression governing the evolution of carbon reads

55 $$\begin{aligned} \frac{\partial y_C}{\partial t}= \frac{\partial }{\partial x}D_c\frac{\partial y_C}{\partial x}, \end{aligned}$$

and the ensuing error-function solution adopts the form

56 $$\begin{aligned} y_C(x,t)=y_{s}-y_{s}\mathrm{erf}\left( \frac{x}{2\sqrt{D_c t}}\right) , \end{aligned}$$

where $y_{s}$ and $y_C(x,t)$ are respectively the carbon concentration on the surface, and at a given point in the bulk at time *t*, when expressed in site-fraction.

When the alloying element in the binary system occupies the regular lattice replacing the atoms of matrix component, both Eqs. (53) and  (55), and the corresponding evolution Eqs. (54) and  (56), would render identical concentration profile at any given instant, as indicated by the conversion relation in Eq. (14). However, since the site- and mole-fraction of components occupying interstitial sublattice are distinctly related (Eq. (56)), similar spatial variation in the concentration profile cannot be expected. In other words, primarily due to the varied descriptions of carbon content, a complete agreement in the concentration profiles rendered by Eqs. (54) and  (56), at time *t*, cannot be inherently assumed. Therefore, in order to explicate the disparity introduced in the carbon distribution exclusively by the different treatment of concentration, the one-dimensional profile emerging from Eqs. (54) and  (56), at a given instant, is comparatively studied.

Site- and mole-fractions are fundamentally different forms of expressing the composition of a multicomponent system. These representations also vary significantly in their respective description, as indicated by Eqs. (9) and  (11). Consequently, the outcomes of evolution Eqs. (54) and  (56) cannot be directly compared in a single framework. To that end, for the comparative analysis, two mole-fraction profiles, referred to as ‘nominal’ and ‘true’ , are defined. While the ‘nominal’ profile pertains to the results of Eq. (54), the outcomes of Eq. (56), which is inherently expressed in site-fraction, is converted to mole-fraction through Eq. (16) for comparison, and is considered as ‘true’ mole-fraction. This ‘true’ mole-fraction, along with ‘nominal’ mole-fraction solution are plotted in Fig. 2. Given the primary focus of the illustration in Fig. 2, the concentration profiles are estimated by assuming $\sqrt{D_c t}=1$. Moreover, in order to realise the effect of stoichiometry, in addition to $a^{\alpha }=3$, a ‘true’ mole-fraction resulting from a different lattice-constant ($a^{\alpha }=9$) is appended to this illustration.

For a surface concentration of $c_s=0.2$, Fig. 2 unravels a noticeable disparity between the ‘true’ mole-fraction profile based on Eq. (56) and error-function solution associated with of ‘nominal’ mole-fraction, Eq. (54). Moreover, it is evident from this illustration that the effect of lattice constant on the concentration profile is rather insignificant, as profiles pertaining to $a^{\alpha }=3$ and 9 visibly overlap. In order to understand the influence of $c_s$ on the disparity between ‘true’ and ‘nominal’ mole-fraction profiles, different surface concentrations have been considered in the current analysis.

**Figure 3:**
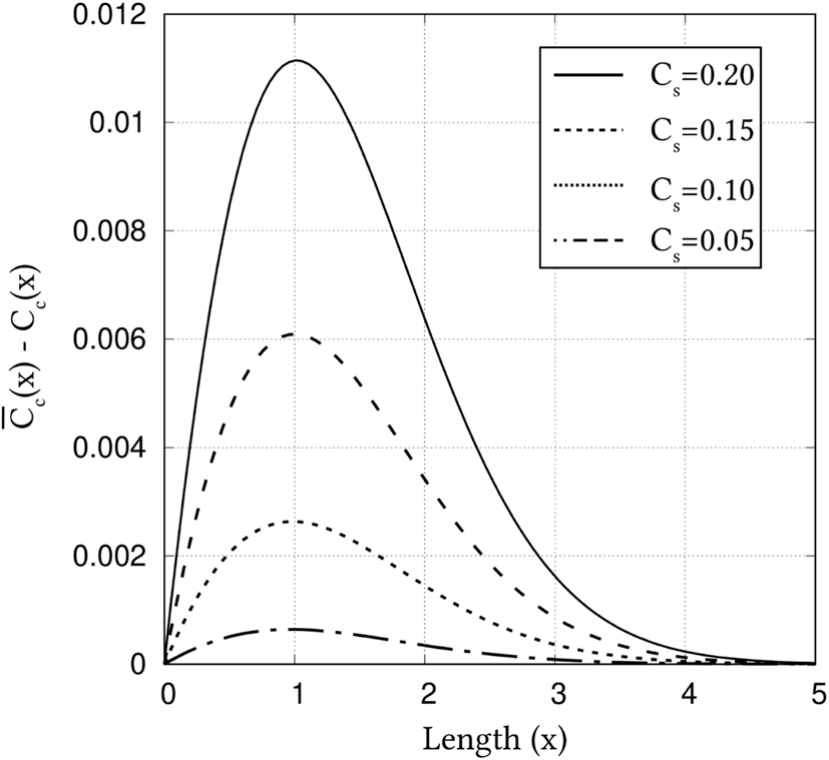
Cumulative influence of surface concentration, and spatial position, on the disparity in the carbon distribution rendered by mole- and site-fraction based formulations in Eqs. (53) and  (55), respectively.

One-dimensional concentration profile resulting from the site- and mole-fraction based error-function solution, for various surface carbon-content, is plotted in Fig. 2. Irrespective of the surface concentration, the profiles pertaining to the ‘true’ mole-fractions do not agree with the ‘nominal’ solutions of Eq. (54). However, it is evident that the surface concentration influences the degree of disparity between the ‘true’ and ‘nominal’ mole-fraction profiles. In other words, Fig. 2 apparently indicates that with decrease in the surface concentration, the difference between the true and nominal profile relatively reduces. Moreover, Fig. 2 reveals that the disparity between the concentration profiles is not constant, but rather depends on spatial position.

In order to cumulative illustrate the influence of surface concentration, and spatial position, on the disparity of the concentration profiles, the difference in the ‘true’ and ‘nominal’ error-function solution, $\bar{c_C}(x)-c_C(x)$, is plotted in Fig. 3. In complete agreement with Fig. 2, this illustration unravels that the disparity in concentration profiles relatively reduces with the decreases in surface concentration, and exhibits a significant spatial-dependency.

## Simulation results and discussion

In order to explicate the ability of the present site-fraction based grand-potential approach to distinguish substitutional and interstitial diffusion accompanying a phase transformation, decomposition of austenite into ferrite is modelled in binary, Fe–C^42^, and ternary, Fe–C–Mn, systems^46,49^. A representative one- and two-dimensional domain with identical length of $84\mu$m is considered for this numerical treatment. However, two-dimensional set-up includes a width of size $10\mu$m.

Irrespective of the dimension, the simulation domains are discretised into identical grids through finite-difference scheme. Each of the equi-sized grids, in this discretisation, is rendered access to six adjacent cells in principal directions. Evolution equations pertaining to phase-field and diffusion (or chemical) potential are explicitly solved over the domain using forward-marching Euler’s scheme in finite-difference framework. While no-flux boundary condition is imposed in one-dimensional setup, periodic and no-flux conditions are respectively considered along the lateral and longitudinal ends of the two-dimensional domain.

**Figure 4:**
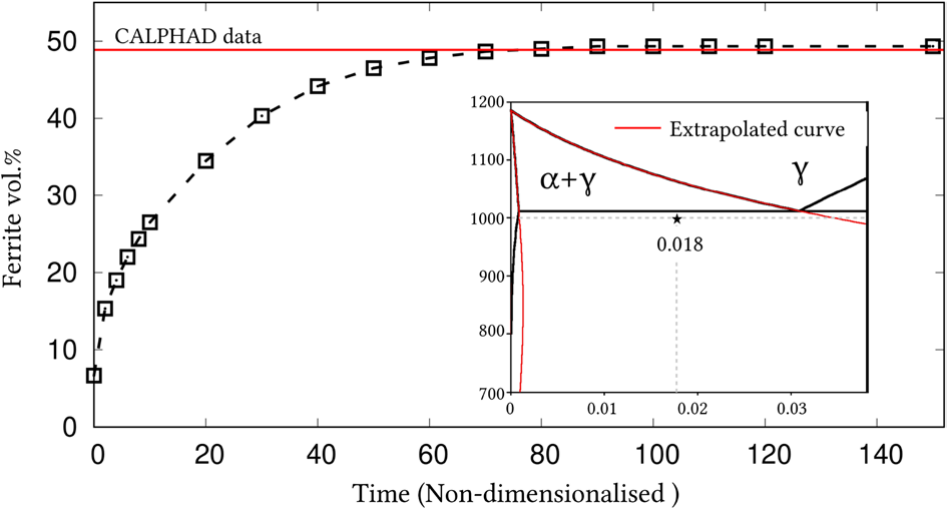
Progressive increase in the volume-fraction of ferrite, with time, which accompanies the austenite decomposition modelled through the present approach. Phase-fraction predicted by CALPHAD is included to verify the quantitative aspect of the treatment. As a subset, phase-diagram with extrapolated curves determined from TcFe8 data is included.

The width of the diffuse interface in the simulation domain is fixed by assigning a definite value to the length-scale parameter, $\epsilon =0.56\mu$m. The interface properties of the present site-fraction based formalism is discussed elsewhere^20^. Relaxation parameter, which ensures the stability of the diffuse interface during the evolution, is considered to be $\tau =1.72\times 10^{9}$Jsm$^{-4}$. Moreover, identical molar-volume of $V_m=7.17 \times 10^{-6}$m$^{3}$mol$^{-1}$ is adopted for both ferrite and austenite, and the energy-density of interface separating these phases is $\gamma =0.49\mu$m$^{-2}$, for both one- and two-dimensional setup. In the subsequent sections, the temporal evolution of phases, concentrations and other variables are discussed based on time *t* which is non-dimensionalised through the factor $t'=\frac{V_m\kappa T}{D_c\gamma L^3}$, where diffusion length of carbon is assumed as $L=0.02$m^50^ and the diffusivity of carbon, $D_c=2\times 10^{-9}\mathrm{m}^2\mathrm{s}^{-1}$^20^.

### Evolution of ferrite in Fe–C system

Austenite to ferrite phase transformation in binary Fe–C system of 0.018 mole-fraction carbon, at 1000 K, is modelled using the present site-fraction based phase-field model in one-dimensional domain. The quantitative driving-force is introduced by formulating the free-energy densities of individual phases based on TCFeC8 database^37^. For fitting the energy-densities with CALPHAD data, numerical approximation technique discussed in section "Energy density description", and a more exhaustively in Ref.^42^, is employed. However, as opposed to the conventional treatment, site-fraction is used to realise the free-energies of the phases, thereby in-keeping with the framework of the present formulation. The temperature-dependent fitting coefficients, which describe the site-fraction based energy-densities of the phases, are tabulated in Table 1.

**Table 1:** Fitting coefficients describing free-energy densities of ferrite and austenite in Fe–C system with 0.018 mole-fraction carbon at 1000 K with $A_{\mathrm{FeC}}^{\Theta }(T)$, $B_{\mathrm{FeC}}^{\Theta }(T)$ and $D_{\mathrm{FeC}}^{\Theta }(T)$ corresponding to the second-, first- and zeroth-order terms^42^, and $\Theta \in \{\alpha ,\gamma \}$.

1000 K	$A_{\mathrm{FeC}}^{\Theta }(T)$ (Jmol$^{-1}$)	$B_{\mathrm{FeC}}^{\Theta }(T)$ (Jmol$^{-1}$)	$D_{\mathrm{FeC}}^{\Theta }(T)$ (Jmol$^{-1}$)
Ferrite ($\alpha$)	47916666.67	5083.33	− 43841.52
Austenite ($\gamma$)	1891666.67	− 78683.33	− 42342.73

**Figure 5:**
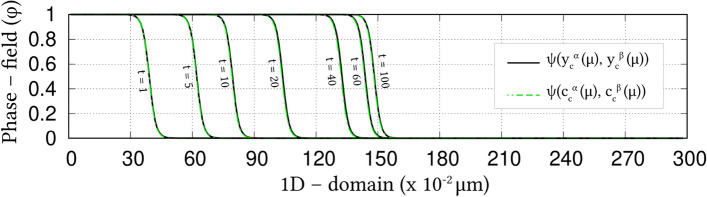
Spatio-temporal evolution of phase-field reflecting the growth of ferrite, in one-dimensional domain, governed by the site-fraction based driving force, which is incorporated through the coefficients in Table 1. Transformation rendered by the conventional mole-fraction based formulation ($\psi (c_{\mathrm{C}}^{\alpha }(\mu ),c_{\mathrm{C}}^{\beta }(\mu ))$), through the coefficients in Table 2, is included for comparison.

The temporal change in the volume-fraction of ferrite is monitored during the phase transformation, and plotted in Fig. 4 with respect to the non-dimensional time, *t*. Equilibrium phase-fraction predicted by the CALPHAD database is included in the illustration to verify the quantitative aspect of the simulation. Moreover, the binary Fe–C phase-diagram with extrapolated curves constructed based on TCFeC8 database, which suggests the equilibrium phase-fraction, is augmented as a sub-plot in Fig. 4.

Consistent with the prediction of CALPHAD database, phase transformation characterised by the progressive increase in ferrite volume-fraction eventually halts when the equilibrium phase-fraction is established in the domain. This agreement in equilibrium phase-fraction is primarily due to the incorporation of the CALPHAD-informed quantitative driving-force through the second-order approximation of the energy-densities. The spatio-temporal evolution of phase-field, which translates into the perceived transformation, is illustrated in Fig. 5. In the initial stages of the evolution, the diffuse interface exhibits a noticeable change in its spatial position, even under a brief duration of time, $t=1$ to $t=5$. However, as the transformation proceeds, the rate of phase-field evolution visibly decreases, until equilibrium phase-fraction is established, when the spatial change completely halts.

**Table 2:** Second-, first- and zeroth-order terms of the mole-fraction based free-energy densities of phases in Fe–C system with 0.018 mole-fraction carbon at 1000 K^42^.

1000 K	${\tilde{A}}_{\mathrm{FeC}}^{\Theta }(T)$ (Jmol$^{-1}$)	${\tilde{B}}_{\mathrm{FeC}}^{\Theta }(T)$ (Jmol$^{-1}$)	${\tilde{D}}_{\mathrm{FeC}}^{\Theta }(T)$ (Jmol$^{-1}$)
Ferrite ($\alpha$)	6354166.67	18837.50	− 43840.96
Austenite ($\gamma$)	203703.70	17550.00	− 43690.85

Identical phase transformation is modelled using the existing mole-fraction based grand-potential approach for a comparative investigation. To that end, in addition to adopting a different framework, the free-energy densities of the individual phases are formulated based on mole-fraction using TCFeC8 CALPHAD database. The fitting coefficients associated with the free-energy densities, wherein concentrations are treated in mole-fraction, are presented in Table 2. The spatio-temporal evolution of phase-field rendered by the conventional mole-fraction based formulation is included in Fig. 5.

**Figure 6:**
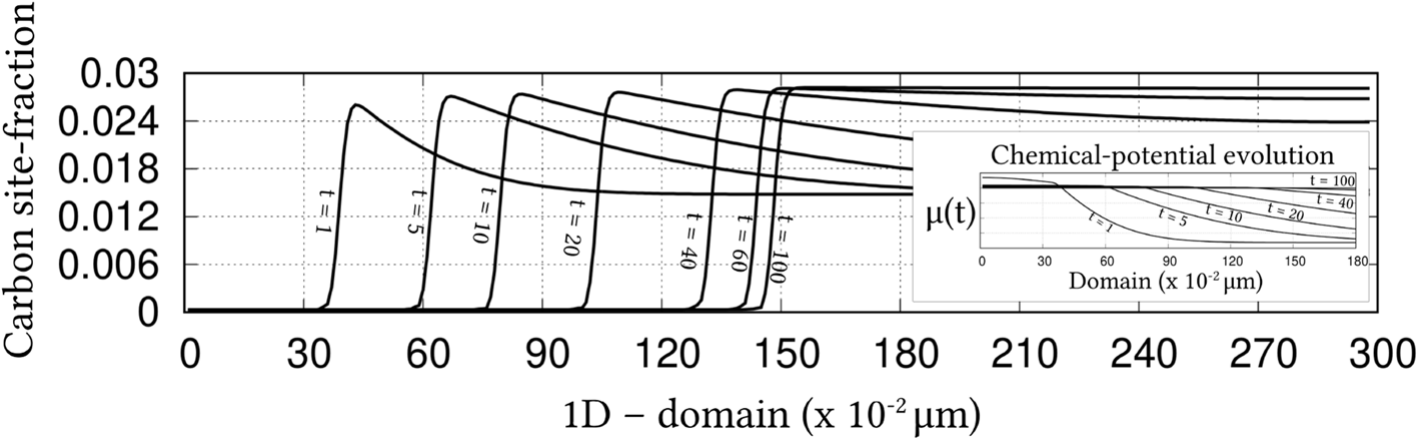
Evolution of carbon concentration, in site-fraction, accompanying the decomposition of austenite. The temporal change in the dynamic variable, *i*.*e* chemical potential of carbon, is included as a subplot.

Despite the different treatment of concentration, Fig. 5 shows that both mole- and site-fraction based models render almost similar spatio-temporal evolution of phase-field. This agreement in the spatial variation of phase-field, at any given time, can be attributed to the quantitative driving-force, which is incorporated in the formulation through appropriately ascertained fitting coefficients. Even though both mole- and site-fraction based formulations yield rather identical phase transformation, it is conceivable that, under equivalent supersaturation, the conventional formulation renders similar the evolution of matrix-species (Fe) for Fe–C and Fe–Mn system. This similarity in the matrix-concentration profile, irrespective of the nature of the system, indicates the inability of the conventional approach to distinguish interstitial and substitutional diffusion. On the other hand, the interstitial-diffusion of carbon accompanying the austenite decomposition is modelled using current site-fraction based grand-potential formalism, and is illustrated in Fig. 6 as time-dependent concentration profiles.

**Figure 7:**
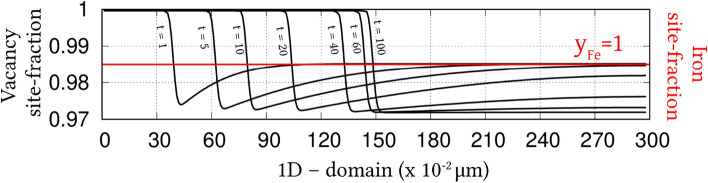
Change in the vacancy concentration with time, owing its reverse diffusion complementing the migration of carbon. The undisturbed site-fraction of matrix-Fe is shown.

The temporal evolution of the fundamental variable, *i*.*e* chemical potential, that dictates the change in the carbon-concentration profile with time, is included as a subplot in Fig. 6. A substantial gradient in the chemical potential, at the early stages of the phase transformation, induces significant diffusion of carbon which is characterised by the visible change in the concentration profile. However, as the potential-gradient progressively decreases, and visibly vanishes, the diffusion halts establishing equilibrium composition in the constituent phases. This interstitial diffusion of carbon in binary Fe–C system is characterised by complementing reverse-migration of vacant site. The spatio-temporal evolution of vacancy site-fraction accompanying interstitial migration of carbon is illustrated in Fig. 7. The ability to model thermodynamically-appropriate migration of interstitial vacant-sites complementing carbon diffusion indicates the effectiveness of the present site-fraction based formulation in handling systems with interstitial lattice. Existing conventional grand-potential model, on the other hand, would have alluded to a rather inaccurate reverse-diffusion of matrix-species. As shown in Fig. 7, the site-fraction of matrix-Fe, under the current formalism, remains unaltered despite the noticeable evolution of the alloying element, and corresponding phase transformation.

### Ferrite growth in ternary Fe–C–Mn alloy

The decomposition of austenite to ferrite, as considered for binary Fe–C system in previous section, is modelled using the present site-fraction based formalism for ternary manganese steel. However, as opposed to one-dimensional domain, a quasi one-dimensional setup with definite width (thus, two-dimensional) is employed for this numerical treatment.

**Figure 8:**
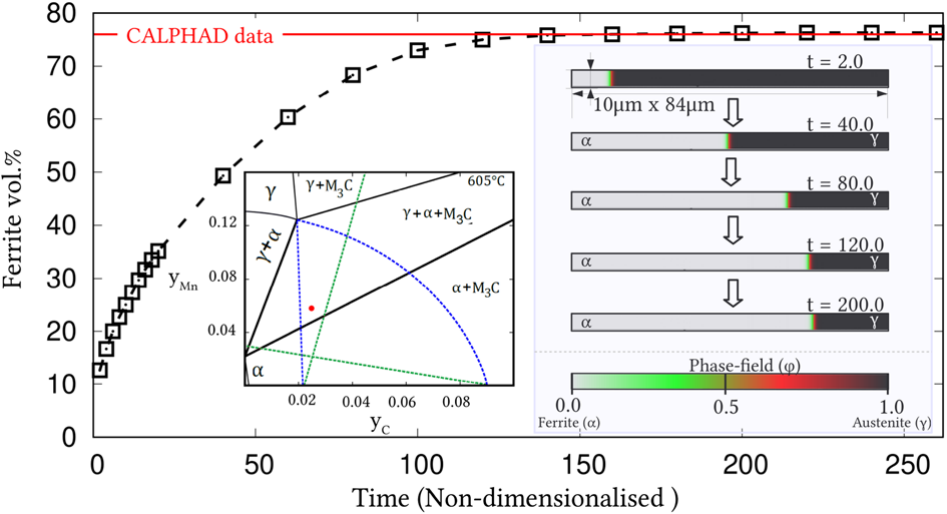
Progressive increase in the volume fraction of ferrite during the austenite decomposition of ternary Fe–C–Mn. As subplots, the isotherm of the ternary alloy at 878 K is included, besides the evolution of phase-field in quasi-one-dimensional domain that correspond to the phase transformation.

In order to model the austenite decomposition at 878 K in ternary steel of 0.025 and 0.042 site-fraction carbon and manganese, respectively, the energy densities of the phases are appropriately formulated based on TCFeC8 and MOBFe3 kinetic database. The fitting coefficients of the site-fraction based free-energy densities, which incorporate quantitative driving-force, are tabulated in Table 3. Furthermore, the entries of interdiffusivity matrix pertaining ternary manganese steel at 878 K is listed in Table 4. The time is non-dimensionalised using diffusivity of manganese for the present discussions, as opposed to $D_c$ in previous section.

**Table 3:** Fitting coefficients describing energy contributions ternary Fe–C–Mn phases where $A_{\mathrm{FeCMn}}^{\Theta }(T)$ and $B_{\mathrm{FeCMn}}^{\Theta }(T)$ respectively denote the second-order terms associated with carbon and manganese, where the corresponding first-order coefficients are $P_{\mathrm{FeCMn}}^{\Theta }(T)$ and $Q_{\mathrm{FeCMn}}^{\Theta }(T)$^20,46,49^.

878 K	$A_{\mathrm{FeCMn}}^{\Theta }(T)$	$B_{\mathrm{FeCMn}}^{\Theta }(T)$	$P_{\mathrm{FeCMn}}^{\Theta }(T)$	$Q_{\mathrm{FeCMn}}^{\Theta }(T)$	$S_{\mathrm{FeCMn}}^{\Theta }(T)$
Ferrite	163210000	190200	− 62240	− 72446	− 36662.02
Austenite	195630	51535	− 22221	− 73082	− 33179.99

**Table 4:** Interdiffusivity matrix governing the evolution rate of diffusion (or chemical) potential at 878 K.

$\varvec{D}_{ik}^{\gamma }$	C	Mn
C	1.31 $\times 10^{-13}$	− 1.47 $\times 10^{-14}$
Mn	1.56 $\times 10^{-21}$	7.71 $\times 10^{-21}$
$\varvec{D}_{ik}^{\alpha }$	C	Mn
C	2.56 $\times 10^{-11}$	− 4.63 $\times 10^{-12}$
Mn	− 4.34 $\times 10^{-19}$	1.17 $\times 10^{-18}$

The increase in the volume-fraction of ferrite, during the phase transformation, is estimated from the simulation domains and illustrated in Fig. 8. In addition to the temporal change in ferrite phase-fraction, this figure shows the two-dimensional domain at different time-steps, which reflects the spatio-temporal evolution of phase-field. Moreover, a section of the ternary Fe–C–Mn isotherm at 878 K ($605^0$C), constructed from TCFeC8 database, depicting the overall composition of the alloy is included as a subplot in Fig. 8. The ternary isotherm shows that the alloy composition, represented by the red dot, lies above the partition - no-partition line. Therefore, the phase transformation is cumulatively governed by the diffusion of both alloying elements, carbon and manganese. Moreover, since the ternary composition lies in the three-phase region of the isotherm, the equilibrium phase-distribution comprises ferrite and austenite.

Simulation domains at various time-steps, which are appended as subplots in Fig. 8, reveal that growth of ferrite halts before consuming austenite completely. In other words, it is evident from the simulation domain that the equilibrium microstructure consists of ferrite and austenite, in complete agreement corresponding isotherm. Additionally, as shown in Fig. 8, the progressive increase in the volume-fraction of ferrite eventually stops at a definite phase-fraction, which is largely coincides with the suggestion of CALPHAD data.

**Figure 9:**
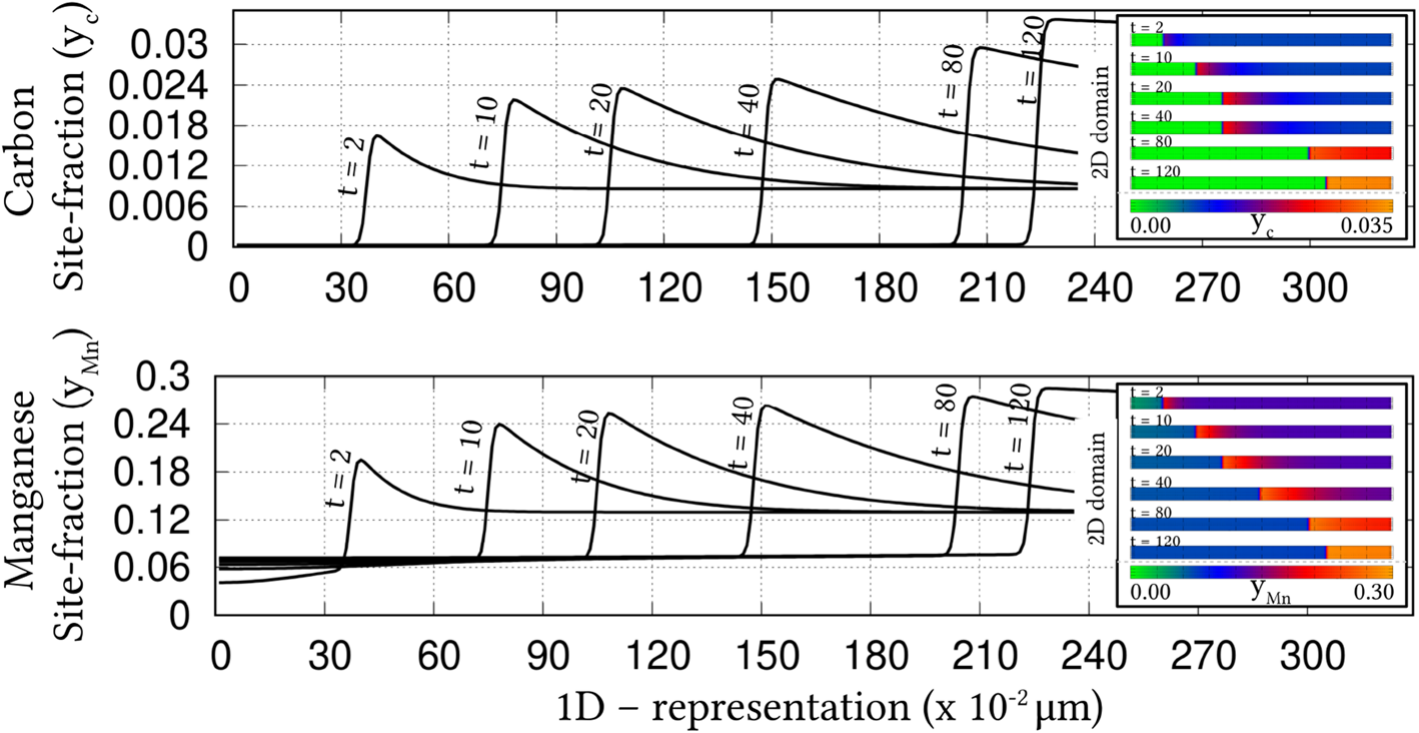
Temporal evolution of site-fraction based one-dimensional concentration profiles of carbon (top) and manganese (bottom) during the austenite decomposition in ternary Fe–C–Mn system. The corresponding distribution of the alloy elements over the entire domain is shown as subplots.

**Figure 10:**
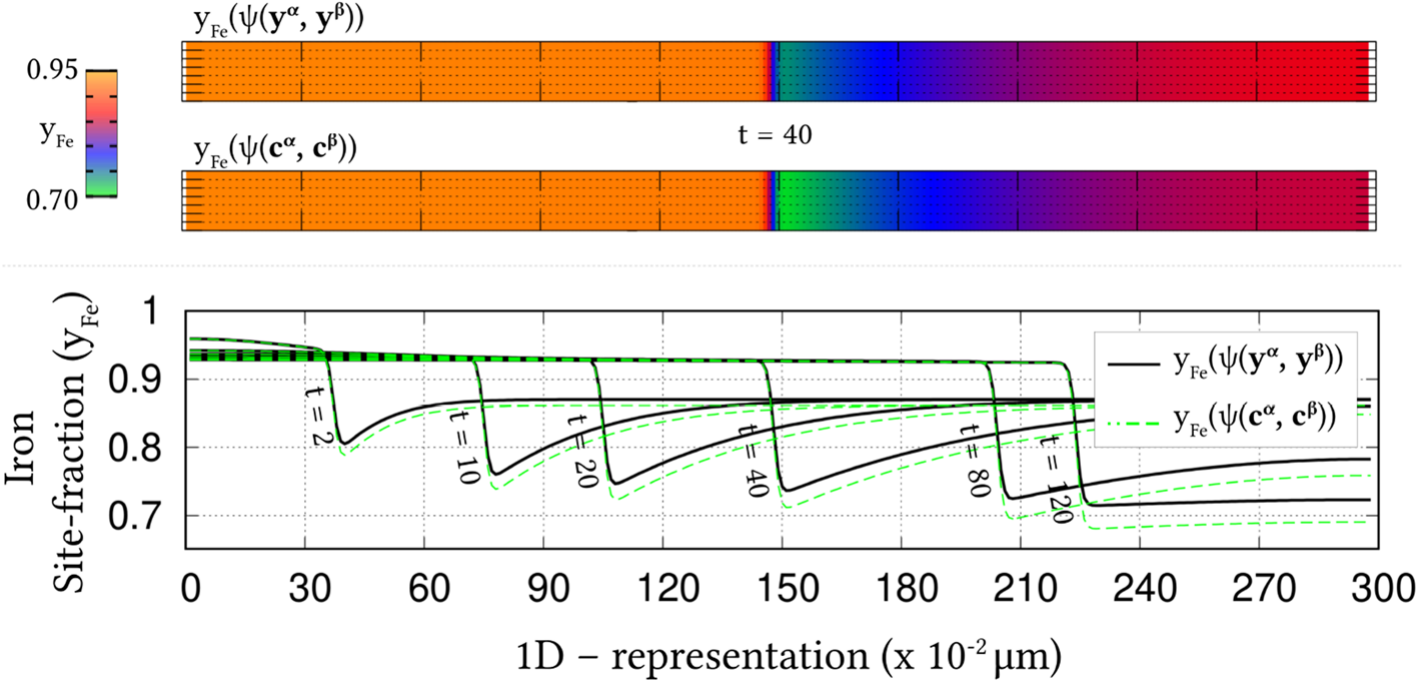
(Top) Difference in the concentration distribution of Fe-matrix owing to the characteristic treatment of concentrations in conventional ($\psi (\varvec{c}^{\alpha }(\mu ),\varvec{c}^{\beta }(\mu ))$) and present ($\psi (\varvec{y}^{\alpha }(\mu ),\varvec{y}^{\beta }(\mu ))$) grand-potential approach. (Bottom) Corresponding disparity in the evolution of one-dimensional concentration profile of matrix chemical-species due to the difference in the handling of alloying elements.

The spatio-temporal evolution of the alloying elements, carbon and manganese, are illustrated in Fig. 9 by plotting the corresponding one-dimensional concentration profiles at different time-steps. Moreover, the change in the distribution of carbon and manganese concentration in the two-dimensional simulation domain, during the austenite decomposition, is augmented as a subplot in Fig. 9. These plots indicate that the gradient in the concentrations, which mirror the appropriate diffusion (or chemical) potential, are substantial in the early stages of the transformation. The spatial disparity in both carbon and manganese concentrations, within the matrix phase, gradually decrease as the evolution proceeds. Ultimately, the gradient disappears and the concentration assumes a definite value within each phase, when system reaches equilibrium. Previous analysis comparing the existing mole-fraction based model to the current site-fraction formalism seemingly suggests that, under equivalent supersaturation and appropriate fitting coefficients, both approaches might yield similar phase-fraction and evolution of concentrations. However, considering that the conventional grand-potential technique fails to accurately capture interstitial diffusion, and alludes to a complementing migration of matrix-species, a disparity in the evolution of Fe is expected. Therefore, the temporal evolution of the one-dimensional matrix-concentration profile rendered by the mole- and site-fraction based approaches are collectively plotted in Fig. 10. Moreover, the concentration distribution of Fe in the two-dimensional domain at $t=40$, emerging from the conventional and current grand-potential formalism, is included in this depiction. Even though the concentration profile pertaining to the conventional model is principally described in mole-fraction, it appropriately converted to site-fraction, through Eq. (14), for a comparative illustration in Fig. 10.

The distribution of matrix-species in the simulation domain, at $t=40$, resulting from the approach wherein concentrations are treated in mole-fraction, is visibly different from the corresponding distribution rendered by present site-fraction based grand-potential model. This disparity in matrix-concentration distribution becomes more evident when temporally-varying one-dimensional Fe-concentration profiles pertaining to the different formulations are presented together as in Fig. 10. The significant difference in Fe-concentration distribution is primarily due to the different approaches adopted by the phase-field techniques to ascertain the dependent matrix-concentration. In conventional formulation, since the description of mole-fractions is exploited to estimate the dependent variable, the evolution of the matrix-species is implicitly coupled to the spatio-temporal changes in all alloying elements, irrespective of their lattice position and the mode of diffusion they adopt. On the other hand, since site-fraction, by definition, separates components of regular and interstitial lattice, the evolution of matrix is coupled with only to those chemical species that occupy the regular lattice. Therefore, by treating components based on their lattice position, the present site-fraction based grand-potential formalism elegantly distinguishes interstitial and substitutional diffusion. This different treatment of dependent matrix-concentration is responsible for the disparity in its concentration profile, as shown in Fig. 9.

### Austenite decomposition to ferrite under paraequilibrium

Besides generally assumed local- or ortho-equilibrium, phase transformations, particularly in multicomponent systems, are governed by other conditions including Negligible-Partitioning Local-Equilibrium (NPLE) and paraequilibrium^51,52^. As opposed to ortho-equilibrium, wherein the phase transformation is governed by the diffusion of all chemical species, under both NPLE and paraequilibrium, the diffusion is restricted to specific alloying elements, generally the faster diffusing ones. A characteristic feature that separates NPLE from paraequilibrium is the presence of a concentration-spike pertaining to slower-diffusing component at the interface^51^. In other words, each of these equilibrium conditions, treat the evolution of alloying elements uniquely. For instance, phase transformation under paraequilibrium is almost exclusively dictated by diffusion of few components, while the rest largely remain immobile during the entire microstructural evolution^53^. As illustrated by the isotherm in Fig. 8, since these equilibrium conditions form an integral aspect of the phase transformation in multicomponent systems, phase-field techniques must be sufficiently sophisticated to handle the characteristic evolution of the alloying elements. However, given that the conventional mole-fraction based formulations fail to distinguish chemical species depending on their lattice positions, the corresponding approaches are inherently inadequate to impose paraequilibrium condition. On the other hand, present formalism by treating concentrations in site-fraction lends itself to model microstructural transformations under paraequilibrium. This ability of the site-fraction based approach is elucidated by handling the Fe–C–Mn alloy, considered in the previous section, under paraequilibrium and comparing the resulting evolution with the outcomes of regular local-equilibrium condition illustrated in Fig. 8.

**Figure 11:**
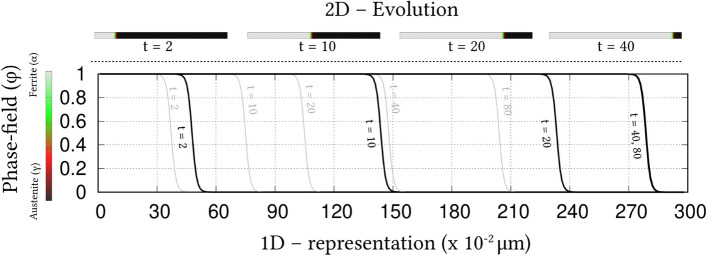
Spatio-temporal evolution of phases in two- and one-dimensional domain (top and bottom, respectively) reflecting austenite decomposition under paraequilibrium. The growth of ferrite under orthoequilibrium in included in one-dimensional plot for comparison.

**Figure 12:**
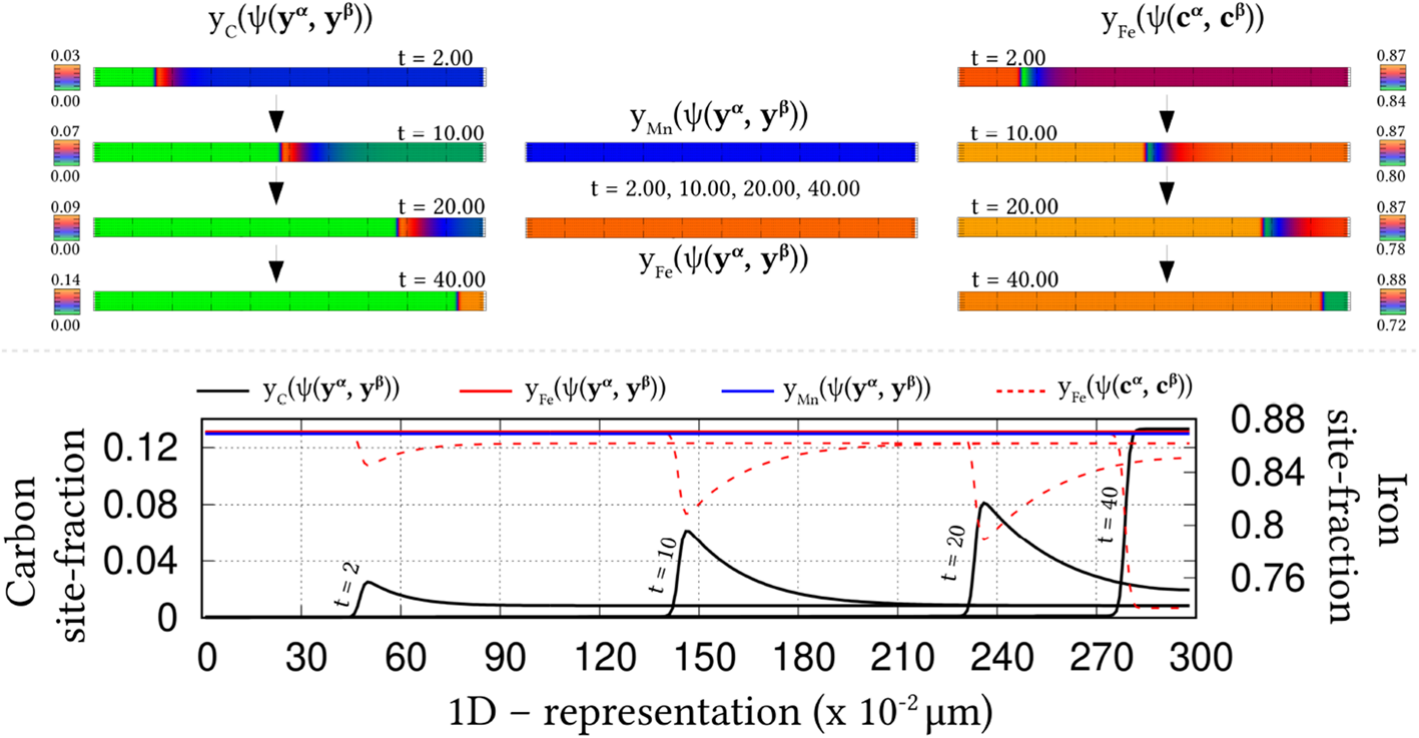
(Top) Respective time-dependent and -independent concentration distribution of carbon, manganese and matrix-species accompanying growth of ferrite under paraequilibrium. Evolution of Fe-concentration distribution, which inaccurately captured by the convention mole-fraction based technique, is included. (Bottom) One-dimensional concentration profiles depicting the evolution of carbon, and time-invariant behaviour of Fe- and Mn-concentrations. Reverse complementing diffusion of Fe-matrix, inherently alluded by the conventional approach ($\psi (\varvec{c}^{\alpha }(\mu ),\varvec{c}^{\beta }(\mu ))$), is illustrated by the change in the Fe-concentration profile.

In ternary system considered in previous section, paraequilibrium is established by eliminating the supersaturation in manganese, and assigning identical values to the corresponding fitting coefficients. The lack of supersaturation, and identical coefficients, ensure that the diffusion potentials of manganese are same in both phases. Consequently, devoid of any driving-force, the manganese concentration remains unaltered, while phases evolve solely governed by the diffusion of carbon. The spatio-temporal evolution of phase-field pertaining to the Fe–C–Mn alloy, under this imposed paraequilibrium condition, is shown in Fig. 11. For comparison, evolution of phase-field under orthoequilibrium condition, as illustrated in subplot of Fig. 8, is included in this illustration. While the difference in the treatment of concentration yields almost similar phase transformation (Fig. 5), the equilibrium conditions by effecting the driving-force influence the rate of phase-field evolution, and equilibrium phase-fraction as shown in Fig. 11. Even though by removing the manganese supersaturation, and assigning identical fitting coefficients, a condition akin to paraequilibrium can be imposed in the existing grand-potential model, the evolution of carbon cannot be decoupled from the complementing spatio-temporal changes in the matrix-species. This characteristic outcome of the mole-fraction based formalism is illustrated by considering concentration distribution of all alloying elements along with matrix-species, and plotting the corresponding one-dimensional concentration profiles in  Fig. 12.

Through appropriate fitting coefficients, and equivalent supersaturation, almost identical distribution of carbon is rendered by both mole- and site-fraction based technique. Similarly, by ensuring that the driving-force, which primarily govern the manganese evolution, are absent, an unperturbed concentration distribution can be maintained all-through the phase transformation in both techniques. However, only the present site-fraction based formalism essentially captures the distribution of matrix-species that accurately relates to paraequilibrium. In other words, during the paraequilibrium governed austenite decomposition, akin to manganese, the matrix-concentration distribution remains unaltered. Owing to the approach adopted to ascertain dependent variable, in mole-fraction based grand-potential model, the Fe-concentration evolves complementing the spatio-temporal changes of carbon. This complementing reverse-diffusion of matrix-components, which renders an inaccurate depiction of paraequilibrium, is evident in both two-dimensional concentration distribution and one-dimensional profile of matrix-Fe, as shown in Fig. 12. Unlike the conventional technique, since the present site-fraction based grand-potential model separates the diffusion of carbon from the evolution any regular-lattice component, particularly the matrix-species, paraequilibrium condition can be accurately introduced in this framework.

## Conclusion

Alloys with high applicability, generally, extend beyond binary composition, and are multicomponent in nature. Chemical species constituting a multicomponent system, owing to their features, might occupy definite positions in the lattice, thereby establishing an atomic arrangement with sublattices. A rather straightforward, but absolutely critical, example of a sublattices can be observed in steels, wherein alloying elements like carbon occupy interstitial sites. In theoretical framework, such systems should be appropriately handled in order to reflect the sublattices associated with them. To that end, in the current work, an existing phase-field model, formulated in grand-potential framework, is re-visited to treat alloy systems which are characterised by components occupying regular and interstitial sites.

Multicomponent phase-field approaches, in general, adopt mole-fraction to describe the concentration of the alloying elements^19,24,25^. When such treatment is extended to handle systems with interstitial components, the undergirding formulation inherently assumes a reverse diffusion of matrix species complementing the migration of elements bound to the interstitial sites. This coupled diffusion of matrix component in response to the evolution of interstitial species contradicts the characteristic feature of the interstitial diffusion. In other words, phase-field technique employing mole-fraction is not sufficiently equipped to distinguish interstitial and substitutional diffusion. The present formalism by expressing concentration in site-fractions, in a computationally-efficient framework, allows for distinct modelling of interstitial and substitutional diffusion by differentiating the components based on their lattice positions. In addition to distinguishing different modes of diffusions, this site-fraction based grand-potential approach facilitates the accurate incorporation of unique equilibrium conditions, like paraequilibrium, which cannot be consistently pursued in a mole-fraction based formulation.

Despite the aforementioned advantages, the present approach is formulated to handle systems with two phases. Consequently, the numerical studies in this work have been confined representative microstructures. Taking cognisance of this restriction, attempts are currently being made to devise a site-fraction based multiphase-field model^47^. Moreover, a grand-potential techniques which could handle complex sublattices, besides regular and interstitial sites, will be reported in the future.

In multicomponent alloys, in addition to the supersaturation of individual chemical-species, a phase transformation is governed by the inherent stresses (or strains) which are characteristically associated with the system. Such additional contributions from the bulk-phases cannot be adequately captured in the current framework. Therefore, in subsequent works, a comprehensive approach with elastic and plastic driving-forces will be presented^13,34^.
